# Association of anthropometry and weight change with risk of dementia and its major subtypes: A meta‐analysis consisting 2.8 million adults with 57 294 cases of dementia

**DOI:** 10.1111/obr.12989

**Published:** 2020-01-03

**Authors:** Crystal ManYing Lee, Mark Woodward, G. David Batty, Alexa S. Beiser, Steven Bell, Claudine Berr, Espen Bjertness, John Chalmers, Robert Clarke, Jean‐Francois Dartigues, Kendra Davis‐Plourde, Stéphanie Debette, Emanuele Di Angelantonio, Catherine Feart, Ruth Frikke‐Schmidt, John Gregson, Mary N. Haan, Linda B. Hassing, Kathleen M. Hayden, Marieke P. Hoevenaar‐Blom, Jaakko Kaprio, Mika Kivimaki, Georgios Lappas, Eric B. Larson, Erin S. LeBlanc, Anne Lee, Li‐Yung Lui, Eric P. Moll van Charante, Toshiharu Ninomiya, Liv Tybjærg Nordestgaard, Tomoyuki Ohara, Toshiaki Ohkuma, Teemu Palviainen, Karine Peres, Ruth Peters, Nawab Qizilbash, Edo Richard, Annika Rosengren, Sudha Seshadri, Martin Shipley, Archana Singh‐Manoux, Bjorn Heine Strand, Willem A. van Gool, Eero Vuoksimaa, Kristine Yaffe, Rachel R. Huxley

**Affiliations:** ^1^ School of Psychology and Public Health La Trobe University Melbourne Victoria Australia; ^2^ Boden Institute of Obesity, Nutrition, Exercise & Eating Disorders University of Sydney Sydney New South Wales Australia; ^3^ The George Institute for Global Health University of Oxford Oxford UK; ^4^ The George Institute for Global Health University of New South Wales Sydney New South Wales Australia; ^5^ Department of Epidemiology Johns Hopkins University Baltimore Maryland USA; ^6^ Department of Epidemiology and Public Health University College London London UK; ^7^ School of Biological & Population Health Sciences Oregon State University Corvallis Oregon USA; ^8^ Department of Biostatistics Boston University School of Public Health Boston Massachusetts USA; ^9^ Department of Neurology Boston University School of Medicine Boston Massachusetts USA; ^10^ Framingham Heart Study Framingham Massachusetts USA; ^11^ The National Institute for Health Research Blood and Transplant Unit in Donor Health and Genomics, Strangeways Research Laboratory University of Cambridge Cambridge UK; ^12^ UK Medical Research Council/British Heart Foundation Cardiovascular Epidemiology Unit, Department of Public Health and Primary Care, Strangeways Research Laboratory University of Cambridge Cambridge UK; ^13^ British Heart Foundation Centre of Excellence, Division of Cardiovascular Medicine Addenbrooke's Hospital Cambridge UK; ^14^ INSERM, U1061, Neuropsychiatry: Epidemiological and Clinical Research University of Montpellier Montpellier France; ^15^ Memory Research and Resources Center, Department of Neurology Montpellier University Hospital Gui de Chauliac Montpellier France; ^16^ Department of Community Medicine and Global Health University of Oslo Oslo Norway; ^17^ Clinical Trial Service Unit, Nuffield Department of Population health University of Oxford Oxford UK; ^18^ Unité INSERM 1219 Université de Bordeaux Bordeaux France; ^19^ INSERM, Bordeaux Population Health Research Center and Department of Neurology Centre Hospitalier Universitaire de Bordeaux Bordeaux France; ^20^ INSERM, Bordeaux Population Health Research Center, UMR U1219 University of Bordeaux Bordeaux France; ^21^ Department of Clinical Biochemistry Rigshospitalet Copenhagen Denmark; ^22^ Department of Clinical Medicine, Faculty of Health and Medical Sciences University of Copenhagen Copenhagen Denmark; ^23^ Department of Medical Statistics LSHTM London UK; ^24^ Department of Epidemiology and Biostatistics, School of Medicine University of California San Francisco San Francisco California USA; ^25^ Department of Psychology, and Centre for Ageing and Health – AgeCap University of Gothenburg Gothenburg Sweden; ^26^ Department of Social Sciences and Health Policy Wake Forest School of Medicine Winston‐Salem North Carolina USA; ^27^ Department of Neurology, Amsterdam UMC University of Amsterdam Amsterdam the Netherlands; ^28^ Institute for Molecular Medicine Finland (FIMM) University of Helsinki Helsinki Finland; ^29^ Department of Public Health University of Helsinki Helsinki Finland; ^30^ Department of Molecular and Clinical Medicine, Sahlgrenska Academy University of Gothenburg Gothenburg Sweden; ^31^ Kaiser Permanente Washington Health Research Institute Seattle Seattle Washington USA; ^32^ Kaiser Permanente Center for Health Research NW Portland Oregon USA; ^33^ Research Institute California Pacific Medical Center San Francisco Carlifornia USA; ^34^ Department of General Practice, Amsterdam UMC University of Amsterdam Amsterdam the Netherlands; ^35^ Department of Epidemiology and Public Health, Graduate School of Medical Sciences Kyushu University Fukuoka Japan; ^36^ Department of Neuropsychiatry, Graduate School of Medical Sciences Kyushu University Fukuoka Japan; ^37^ Faculty of Science University of New South Wales Sydney New South Wales Australia; ^38^ Neuroscience Research Australia Sydney New South Wales Australia; ^39^ Faculty of Medicine Imperial College London London UK; ^40^ OXON Epidemiology London UK; ^41^ Department of Neurology, Donderds Centre for Brain, Behaviour and Cognition Radboud University Medical Center Nijmegen the Netherlands; ^42^ Sahlgrenska University Hospital Östra Sjukhuset Gothenburg Sweden; ^43^ Glenn Biggs Institute for Alzheimer's and Neurodegenerative Diseases University of Texas Health Sciences Center San Antonio Texas USA; ^44^ INSERM U1153 Hôpital Hôtel‐Dieu Paris France; ^45^ Department of Chronic Diseases and Ageing Norwegian Institute of Public Health Oslo Norway; ^46^ Norwegian National Advisory Unit on Aging and Health Vestfold Hospital Trust Tønsberg Norway; ^47^ Department of Geriatric Medicine Oslo University Hospital Oslo Norway; ^48^ Faculty of Medicine University of Oslo Oslo Norway; ^49^ Department of Psychiatry University of California San Francisco San Francisco California USA; ^50^ College of Science, Health and Engineering La Trobe University Melbourne Victoria Australia; ^51^ Faculty of Health Deakin University Melbourne Victoria Australia

## Abstract

Uncertainty exists regarding the relation of body size and weight change with dementia risk. As populations continue to age and the global obesity epidemic shows no sign of waning, reliable quantification of such associations is important. We examined the relationship of body mass index, waist circumference, and annual percent weight change with risk of dementia and its subtypes by pooling data from 19 prospective cohort studies and four clinical trials using meta‐analysis. Compared with body mass index–defined lower‐normal weight (18.5‐22.4 kg/m^2^), the risk of all‐cause dementia was higher among underweight individuals but lower among those with upper‐normal (22.5‐24.9 kg/m^2^) levels. Obesity was associated with higher risk in vascular dementia. Similarly, relative to the lowest fifth of waist circumference, those in the highest fifth had nonsignificant higher vascular dementia risk. Weight loss was associated with higher all‐cause dementia risk relative to weight maintenance. Weight gain was weakly associated with higher vascular dementia risk. The relationship between body size, weight change, and dementia is complex and exhibits non‐linear associations depending on dementia subtype under scrutiny. Weight loss was associated with an elevated risk most likely due to reverse causality and/or pathophysiological changes in the brain, although the latter remains speculative.

**Abbreviations**
BMI: body mass index
CI: confidence intervals
HR: hazard ratio
WC: waist circumference

## 1 INTRODUCTION

Dementia, a disease primarily of aging, affects an estimated 47 million people globally.^1^ It is a heterogeneous condition chiefly comprising Alzheimer disease (60‐70% of cases) and vascular dementia accounting for about 15% of cases, although the two subtypes frequently co‐occur.^1^, ^2^ Aging, family history, and sarcopenia are important risk factors for dementia, and there is growing evidence that vascular risk factors, such as diabetes, may also confer increased risk, particularly for vascular dementia, although findings are inconsistent.^3^, ^4^

Excess body weight, typically defined as having a high body mass index (BMI), has been causally linked to a large number of chronic conditions, particularly vascular disease.^5^ While the relationship between BMI and dementia has been the subject of several large‐scale epidemiological studies, the findings have been inconsistent: some studies have reported that only individuals at the extreme ends of the body size spectrum (ie, underweight and obese) experience an increased dementia risk,^6^, ^7^ while others have documented positive^8^ and even inverse^9^ associations. Measures of central obesity, such as waist circumference (WC), have been argued to be more informative measures of obesity‐related risk compared with BMI. Currently, little is known about the association between central obesity and dementia risk.

As nonvascular and vascular dementia have different pathophysiologies, any association with body size may similarly differ according to endpoint. Distinguishing between *possible* dementia subtypes in any analysis with measures of body size may, therefore, prove informative in explaining some of the observed heterogeneity. Further, whether sex differences exist, or if the association between body size and dementia risk differs in middle and later life remains unclear.^10^, ^11^

In this meta‐analysis, we examined the relationships of body size with all‐cause dementia and possible vascular and nonvascular dementia by sex and baseline age in participants free of dementia who had their body size assessed at baseline and were later followed up on dementia status. Using repeat measures, where available, we explored the association between standardized annual weight change during follow‐up with subsequent dementia risk.

## 2 METHODS

The investigators of studies that were either identified from previous systematic reviews and meta‐analyses,^6^, ^7^, ^12^, ^13^, ^14^ or who were known to Dementia Pooling Project^15^ collaborators, were contacted and asked to contribute results for their studies (Figure S1). Seven of the 14 studies from the Dementia Pooling Project contributed data that were included in the analyses. Additional relevant data from 28 studies were identified from five previous systematic reviews and meta‐analyses of the association. Fourteen studies that had not contributed to previous overviews were identified through a PUBMED search by one investigator (CMYL). This was limited to human subjects and the period up to 1 September 2017, based on the search strategy: ([body mass index OR body weight OR obesity OR waist circumference] AND dementia) OR (clinical trial AND incident dementia). Studies were deemed eligible for inclusion if BMI or WC were collected at baseline and dementia status was available at follow‐up. Subsequently, 42 invitations were issued inviting study investigators to collaborate in this pooling study. Of these, 10 studies contributed results. Collaborators provided information on a further eight studies that had not been identified by the literature search, or which had not been included in previous overviews, as the data were unpublished; six of these studies provided data. A total of 23 studies responded (n = 2 790 753).

BMI was calculated as weight in kilogrammes divided by height in metres squared. Directly measured (n = 2 760 602; 98.9%) and self‐report (n = 30 151; 1.1%) height and weight were used in the calculation. WC was measured either at the midpoint between rib and iliac crest (n = 217 051; 29.9%), narrowest waist (n = 9768; 1.7%), umbilicus (n = 12 428; 1.3%), or narrowest waist or umbilicus (n = 486 275; 67.0%). Annual percent weight change was calculated as follows:

$$\frac{100\times \left(\text{last body weight measure prior to dementia follow}\;\mathrm{up}-\text{baseline body weight measure}\right)}{\text{baseline body weight measure}}÷\frac{\left(\text{date}\;\mathrm{at}\;\text{last body weight measure}-\text{date}\;\mathrm{at}\;\text{baseline body weight measure}\right)}{365.25}.$$

Five BMI categories were distinguished: underweight: less than 18.5 kg/m^2^; lower‐normal weight: 18.5‐22.4 kg/m^2^; upper‐normal weight: 22.5‐24.9 kg/m^2^; overweight: 25.0‐29.9 kg/m^2^; and obese: greater than or equal to 30.0 kg/m^2^.

Dementia endpoints were defined by investigators of each individual study. Ascertainment of dementia was by medical examination in 12 studies (Table 1). These studies classified dementia based solely on the Diagnostic and Statistical Manual of Method Disorders criteria^38^, ^39^ or in combination with the National Institute of Neurological and Communication Disorders and Stroke and the Alzheimer's Disease and Related Disorders Association criteria for Alzheimer disease^40^ and the National Institute of Neurological Disorders and Stroke and the Association Internationale pour la Recherche et l'Enseignement en Neurosciences criteria for vascular dementia.^41^ Other methods used in dementia ascertainment included health records (two studies), death records (five studies), and death and health or hospitalization records (four studies). Ten of these studies that used other methods to ascertain dementia classified the disorder based on the International Classification of Diseases and one study used Read Codes^42^ to classify dementia (Table 1).

**Table 1:** Characteristics of included studies

Study	Country	Baseline Year	Baseline Age (years)	N (% female)	Dementia Cases	Dementia Ascertainment	Dementia Criteria	Anthropometric Measurement (waist protocol)
Adult Changes in Thought study^16^	USA	1994‐1996	≥65	4343 (58.3)	1096	Medical examination	DSM‐IV, NINCDS‐ADRDA	Measured (narrowest waist)
Action in Diabetes and Vascular Disease Preteraz and Diamicron MR Controlled Evaluation trial^17^	International	2001‐2003	55‐88	11 136 (42.5)	109	Medical examination	DSM‐IV	Measured (midpoint between rib and iliac crest)
Aging Multidisciplinary Investigation (AMI) cohort^18^	France	2007	≥65	563 (38.7)	65	Medical examination	DSM‐III‐R, NINCDS‐ADRDA, NINDS‐AIREN	Measured (midpoint between rib and iliac crest)
The Copenhagen City Heart Study^19^	Denmark	1976‐1978	≥20	9037 (55.7)	969	Health record	ICD‐8, ICD‐10	Measured (umbilicus)
Cache County Memory Study^20^	USA	1995	≥65	3185 (57.4)	507	Medical examination	DSM‐III‐R, NINCDS‐ADRDA, NINDS‐AIREN	Self‐report
The Copenhagen General Population Study^21^	Denmark	2003	≥20	10 4506 (55.2)	1906	Health record	ICD‐8, ICD‐10	Measured (midpoint between rib and iliac crest)
Clinical Practice Research Datalink^9^	UK	1992‐2007	≥40	19 58191 (54.8)	45507	Health and death record	Read Codes	Measured
Finnish Twin Cohort^22^	Finland	1975	≥18	25 814 (51.1)	960	Death record	ICD‐8, ICD‐9, ICD‐10	Self‐report
Framingham Heart Study^23^	USA	1992‐1996, 1998‐2001	≥60	2232 (56.0)	289	Medical examination	DSM‐IV, NINCDS‐ADRDA, NINDS‐AIREN	Measured (umbilicus)
General Post Office Study^24^	UK	1966‐1967	35‐70	1385 (37.0)	18	Death record	ICD‐8, ICD‐9, ICD‐10	Measured
Hisayama Study^25^	Japan	1988	≥60	1192 (58.3)	350	Medical examination	DSM‐III‐R, NINCDS‐ADRDA, NINDS‐AIREN	Measured (umbilicus)
Health Survey for England and Scottish Health Survey^26^	UK	1995‐2008	16‐99	90 685 (54.7)	524	Death record	ICD‐9, ICD‐10	Measured (midpoint between rib and iliac crest)
Hypertension in the Very Elderly Trial^27^	International	2000	≥80	3337 (60.4)	263	Medical examination	DSM‐IV	Measured
Norwegian Counties Study^28^	Norway	1974‐1978	35‐50	40 978 (50.6)	1173	Death record	ICD‐9, ICD‐10	Measured
Origins of Variance in the Old‐Old^29^	Sweden	1963	45‐65	1152 (69.0)	312	Health record and interview	DSM‐III‐R, NINCDS‐ADRDA, NINDS‐AIREN	Self‐report
Prevention of Dementia by Intensive Vascular Care trial^30^	The Netherlands	2006‐2009	70‐78	3526 (54.4)	233	Medical examination	DSM‐IV	Measured (midpoint between rib and iliac crest)
Primary Prevention Study^31^	Sweden	1970‐1973	45‐55	7394 (0)	788	Death and hospitalization records	ICD‐8, ICD‐9, ICD‐10	Measured
The Perindopril Protection Against Recurrent Stroke Study^32^	International	1995‐1997	26‐91	5865 (29.7)	380	Medical examination	DSM‐IV	Measured
Study of Osteoporotic Fractures^33^	USA	1986‐1988	≥65	1019 (100)	232	Medical examination	DSM‐IV	Measured (narrowest waist)
Three City Study^34^	France	1999‐2000	≥65	6721 (61.4)	832	Medical examination	DSM‐IV	Measured (midpoint between rib and iliac crest)
UK Biobank^35^	UK	2006‐2010	39‐74	48 6275 (54.6)	344	Death and hospitalization records	ICD	Measured (narrowest waist or umbilicus)
Whitehall I Study^36^	UK	1967‐1969	40‐69	17 167 (0)	288	Death record	ICD‐8, ICD‐9, ICD‐10	Measured
Whitehall II Study^37^	UK	1985‐1988	35‐55	5050 (28.3)	149	Death and hospitalization records	ICD‐10	Measured (narrowest waist)

Abbreviations: DSM‐III, Diagnostic and Statistical Manual of Mental Disorders third edition criteria; DMS‐III‐R, Diagnostic and Statistical Manual of Mental Disorders third edition revised criteria; DSM‐IV, Diagnostic and Statistical Manual of Mental Disorders fourth edition criteria; ICD‐8, International Classification of Diseases eighth revision; ICD‐9, International Classification of Diseases ninth revision; ICD‐10, International Classification of Diseases tenth revision; and Related Health Problems; NINCDS‐ADRDA, National Institute of Neurological and Communication Disorders and Stroke and the Alzheimer's Disease and Related Disorders Association criteria; NINDS‐AIREN, National Institute of Neurological Disorders and Stroke and Association Internationale pour la Recherche et l'Enseignement en Neurosciences criteria;

### 2.1 Data analysis

Sex‐specific hazard ratios (HRs) and 95% confidence intervals (CIs) were obtained for all‐cause dementia in relation to (a) each of five BMI categories, with lower‐normal weight as the referent group; (b) each fifth of WC, with the first fifth as the referent group; and (c) each of three annual percent weight change categories (greater than or equal to 0.5% annual weight loss, less than 0.5% annual weight change, and greater than or equal to 0.5% annual weight gain), with less than 0·5% annual weight change as the referent group. Following a pre‐specified common analytic protocol, effect estimates were adjusted for age (model 1); age, smoking, and education or socio‐economic status (model 2; which was the primary model—used in the reporting of outcomes herein); and age, smoking, education or socio‐economic status, diabetes, systolic blood pressure, total cholesterol, blood pressure–lowering medication, cholesterol‐lowering medication, and glucose‐lowering medication where available (model 3). For studies with information on possible dementia subtype, study‐specific estimates were requested for possible vascular dementia and possible nonvascular dementia.

A random effects meta‐analysis was used to combine study‐specific log HR to obtain an overall summary estimate and associated 95% CIs for BMI and WC in relation to all dementia endpoints investigated. Analyses were conducted for women and men combined and then separately. Heterogeneity between studies was quantified using the *I* ^2^ statistic.

Sensitivity analyses were conducted by excluding the largest study and by excluding the studies that did not calculate BMI using objective measures of height and weight. To assess the potential effect of reverse causality, in the same studies, we compared data that were non‐left censored with those that were. As the studies varied by the length of follow‐up, maximum periods of left censoring requested (3, 5, or 10 years) differed between studies. We also compared the associations by study design, by method of dementia ascertainment, and by study baseline mean age. All statistical analyses were performed using Stata/SE 14.0 (Stata Corp LP., USA).

## 3 RESULTS

Results from 23 studies comprised 2 790 753 participants (54% women) in whom 57 294 cases of all‐cause dementia were accumulated during a mean of 9.6 years of disease surveillance. Of these cases, 6792 were classed as nonvascular dementia, and 1214 were recorded as vascular dementia. Of the studies included, information from 14 cohort studies (n = 728 959; 26.1%) were previously unpublished, and four were from clinical trial populations (n = 23 864; 0.9%). Most studies were from Europe (n = 2 758 444; 98.8%), with one study^9^ contributing more than 70% of participants (79% cases of all‐cause dementia). Study characteristics are shown in Tables S1 and S2. In brief, mean age at baseline ranged from 36 to 83 years in men and 37 to 84 years in women. Mean duration of follow‐up varied from 4 to 38 years, with an overall median of 9 years. Mean BMIs at study baseline were 21.9 to 28.2 kg/m^2^ in men and 22.4 to 28.8 kg/m^2^ in women; for WC, the corresponding results were 96.9 to 100.7 and 80.7 to 97.5 cm.

**Figure 1:**
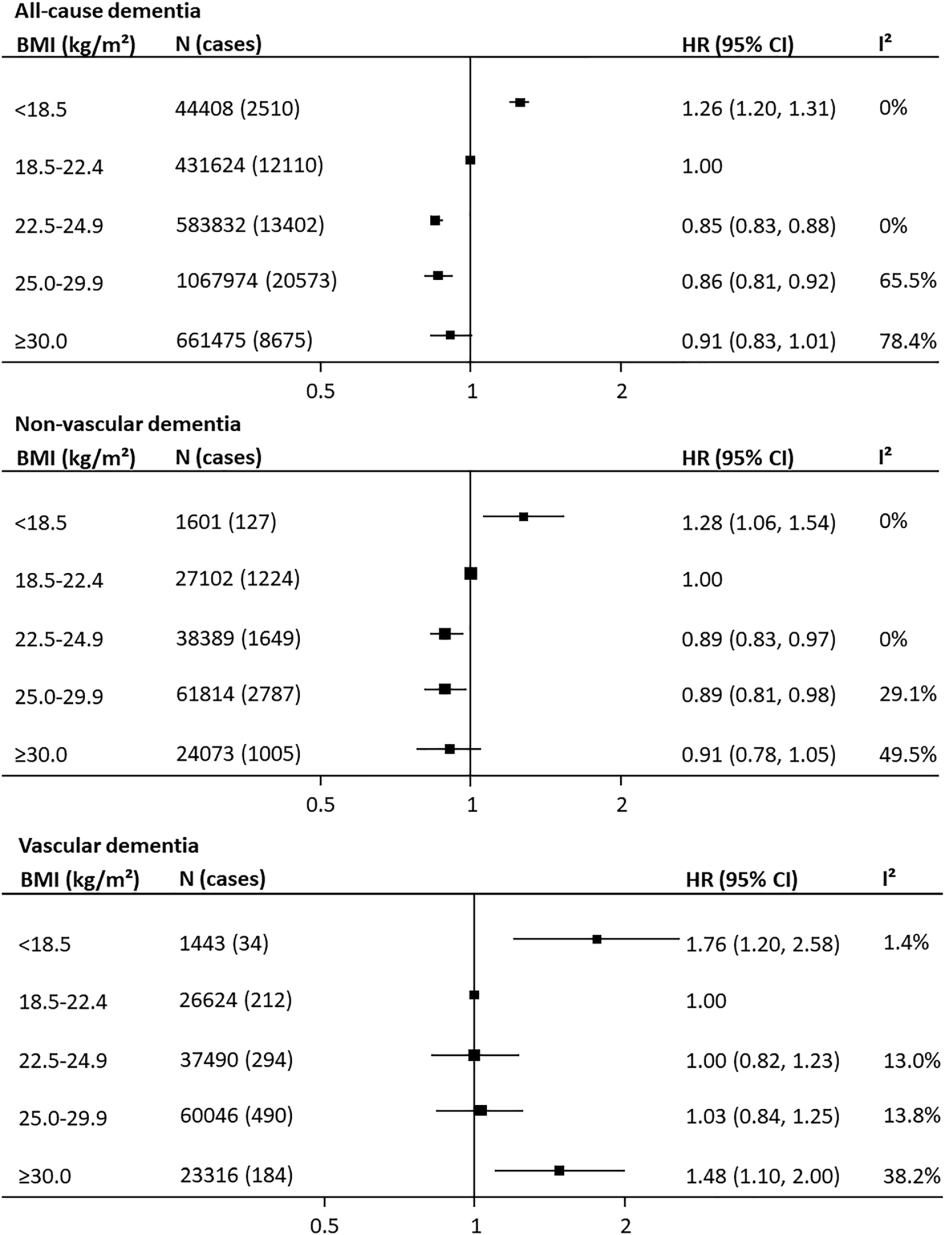
Association between body mass index (BMI) and incident fatal and nonfatal dementia and its major subtypes. Hazard ratios (HRs) and 95% confidence intervals (CIs) adjusted for age, smoking, and education or socio‐economic status

A non‐linear association between BMI with all‐cause dementia was observed: compared with the referent group (BMI: 18.5‐22.4 kg/m^2^) those who were underweight had a one‐quarter greater risk of dementia (HR: 1.26, 95% CI, 1.20‐1.31; Figure 1). This association remained similar after excluding the first 3, 5, or 10 years (median 10 years) of follow‐up in a subset of studies (1.35 [1.24‐1.46]; Figure S2a; Table S3). Relative to the referent group, individuals with upper‐normal BMI, overweight or obesity had a 10% to 15% lower dementia risk (Figure 1). Similar results were obtained after adjustment for age (Figures S3‐S6). Additional adjustment for cardiometabolic risk factors did not materially alter the relationship (model 3; Table S4). Neither were results significantly different in a range of sensitivity analyses (Table S5; Figure S7). Further, the associations were comparable between studies with baseline mean age younger than 60 and 60 years and older (Figure S8) and before and after exclusion of the first 3, 5, or 10 years (median 10 years; Figure S2a; Table S3).

**Figure 2:**
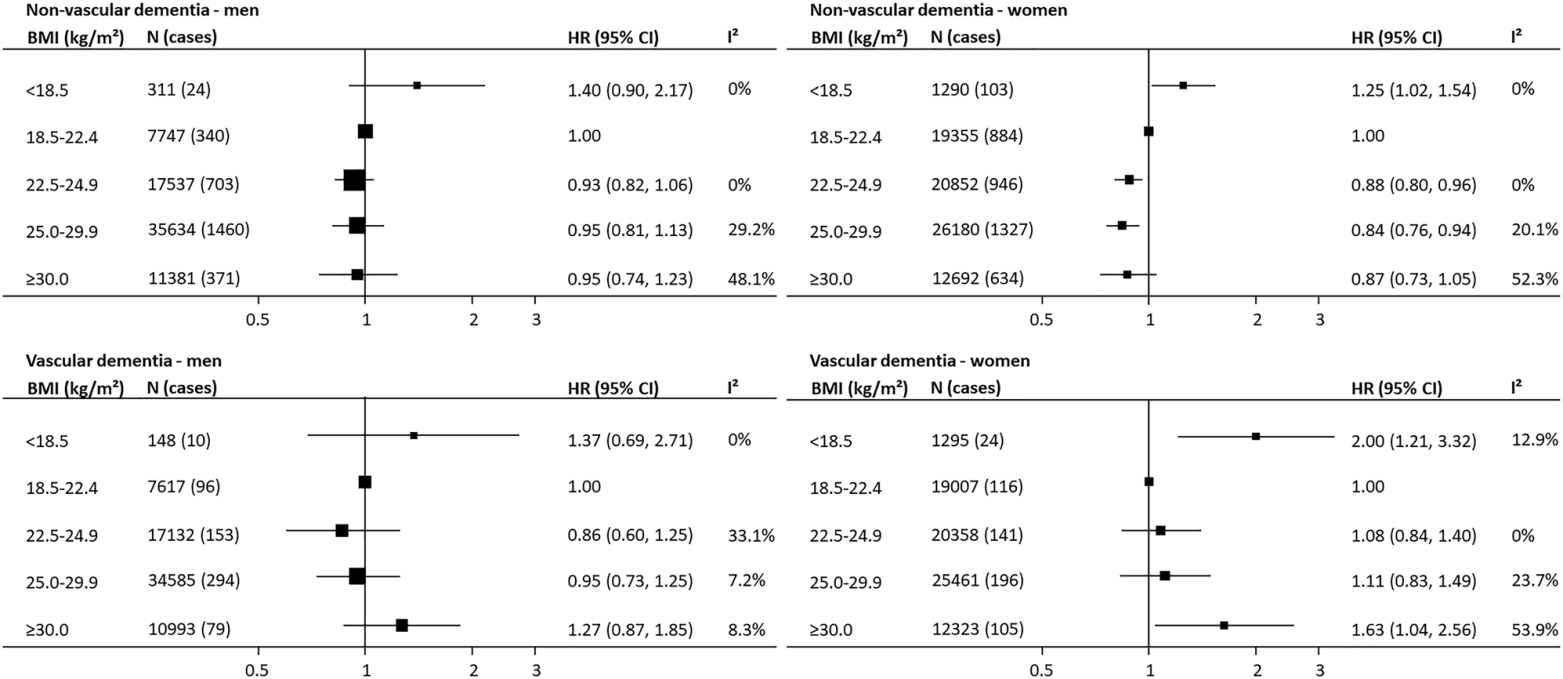
Associations between body mass index (BMI) and incident fatal and nonfatal dementia subtypes by sex. Hazard ratios (HRs) and 95% confidence intervals (CIs) adjusted for age, smoking, and education or socio‐economic status

As with all‐cause dementia, the association between BMI and nonvascular dementia risk was non‐linear. Relative to lower‐normal BMI, individuals categorized as underweight were at approximately one‐quarter increased dementia risk (1.28 [1.06‐1.54]; Figure 1; Table S4). Adjustment for cardiometabolic risk factors did not materially affect the association (Table S4). The findings were similarly robust when restricting the analysis to studies that used measured height and weight. Findings were also consistent between clinical trials and observational studies and between studies with baseline mean age younger 60 and 60 years and older (Figures S8 and S9). There was no evidence of a sex difference in the association (Figure 2). Relative to the referent group, individuals with upper‐normal BMI, overweight, or obesity had 10% lower dementia risk (Figure 1). After excluding the first few years of follow‐up (median 7.5 years), the increased risk of nonvascular dementia in the underweight category remained, but the association was no longer significant in the overweight and obese categories (Figure S2b; Table S3).

Both individuals with underweight or obesity were at increased vascular dementia risk. Compared with the referent group, those who were underweight had an approximate 80% greater dementia risk, and for those in the obese category, the risk was approximately 50% higher (Figure 1). The excess risk among the obese group was largely mediated by cardiometabolic risk factors (HR for model 3: 1.26 [0.87‐1.84]; Table S4). Sensitivity analyses indicated findings compatible with the main result (Figure S9). There was no evidence of a sex difference in the association between BMI and vascular dementia risk (Figure 2). The association was broadly similar in studies with baseline mean age younger than 60 and 60 years and older (Figure S8). Excluding the first few years of follow‐up (median 7.5 years) did not materially influence the relationship (Figure S2c; Table S3).

**Figure 3:**
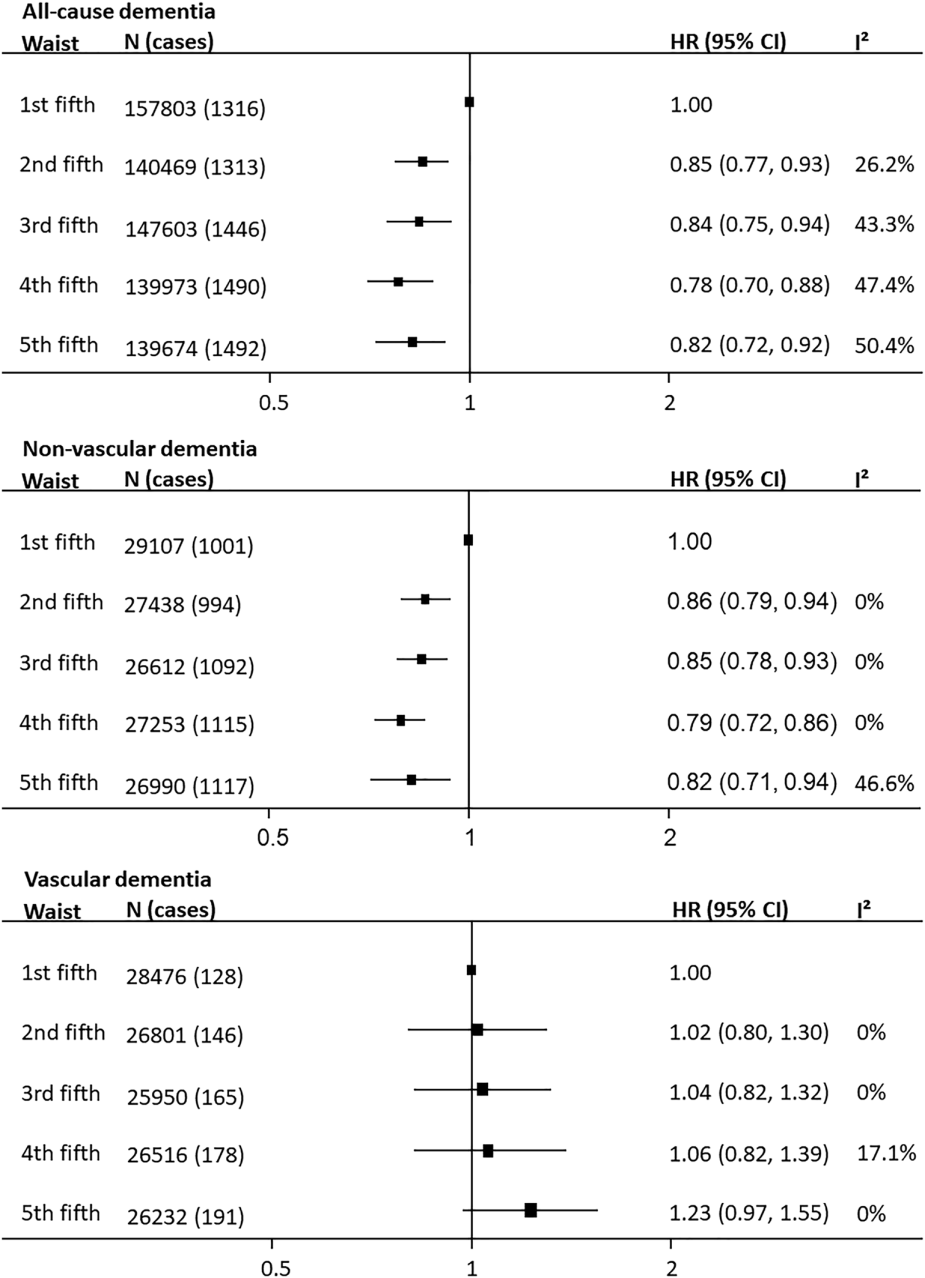
Association between waist circumference and incident fatal and nonfatal dementia and its major subtypes. Hazard ratios (HRs) and 95% confidence intervals (CIs) adjusted for age, smoking, and education or socio‐economic status

A non‐linear association was observed between WC and all‐cause dementia among the 13 studies (725 522 individuals; 54.5% women; 7057 cases) that contributed to the analysis. Compared with individuals in the lowest fifth of WC, individuals with larger WC had 15% to 22% lower all‐cause dementia risk (Figure 3). Similar results were obtained when adjusted for age or after adjustment for cardiometabolic risk factors (Figures S10‐S13; Table S6). The estimates tended to be larger for studies that used death records to ascertain dementia status rather than those that used medical examination (Figure S14). Data from clinical trials produced similar results to those from nontrial populations (Figure S15). The estimate of effect was more pronounced for studies with baseline mean age younger than 60 than 60 years and older especially at higher WC categories (Figure S16).

**Figure 4:**
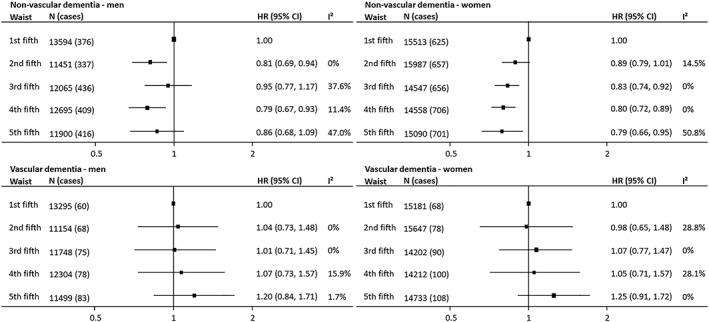
Associations between waist circumference and incident fatal and nonfatal dementia subtypes by sex. Hazard ratios (HRs) and 95% confidence intervals (CIs) adjusted for age, smoking, and education or socio‐economic status

Pooling data from ten studies (5319 nonvascular dementia cases) indicated that, compared with the lowest fifth, all WC categories were associated with lower nonvascular dementia risk (Figure 3; Table S6). Adjustment for cardiometabolic risk factors did not alter the association (Table S6). Data from clinical trials produced a similar pattern (Figure S15), and there was little evidence of a sex difference in the association (Figure 4).

For vascular dementia (808 cases), compared with the referent category, individuals in the highest fifth of WC had roughly one‐quarter higher risk (1.23 [0.97‐1.55]) that was attenuated after further adjustment for cardiometabolic risk factors (Figure 3; Table S6). No sex difference was evident (Figure 4). Data from clinical trial participants contained too few cases to draw meaningful conclusions (Figure S15). Only the highest WC category in studies with baseline mean age 60 years and older was associated with increased vascular dementia risk (Figure S16).

In the analysis of weight change (111 620 individuals; 44.8% female; 5626 dementia cases), compared with individuals who maintained a relatively stable weight during follow‐up, individuals with greater than or equal to 0.5% annual weight loss had approximately one‐third greater all‐cause dementia risk (1.32 [1.18‐1.47]; Table 2). In contrast, greater than or equal to 0.5% annual weight gain was not associated with dementia risk (1.00 [0.89‐1.12]; Table 2). The results remained unchanged for models 1 and 3, and when studies were stratified by baseline mean age (Figures S17‐S19; Table 2). For greater than or equal to 0.5% annual weight loss, the estimate of effect was more pronounced for studies that ascertained dementia by medical examination than studies that used death records (Figure S20).

**Table 2:** Adjusted hazard ratios (HR) with 95% confidence intervals (CI) of dementia by annual percent weight change

Dementia Type		≥0.5% Weight Loss per Year	Reference	≥0.5% Weight Gain per Year
All‐cause dementia, 15 studies				
	N (cases)	23 590 (1668)	44 425 (2254)	43 605 (1704)
Model 2^a^	HR (95% CI)	1.32 (1.18‐1.47)	1.00	1.00 (0.89‐1.12)
	*I* ^2^ (%)	46.2		54.0
Model 3^b^	HR (95% CI)	1.28 (1.15‐1.42)	1.00	0.99 (0.89‐1.11)
	*I* ^2^ (%)	41.1		48.8
Nonvascular dementia, 10 studies				
	N (cases)	8106 (898)	10 929 (932)	9926 (652)
Model 2^a^	HR (95% CI)	1.41 (1.19‐1.67)	1.00	0.99 (0.83‐1.19)
	*I* ^2^ (%)	51.2		51.3
Model 3^b^	HR (95% CI)	1.36 (1.15‐1.61)	1.00	0.98 (0.81, 1.17)
	*I* ^2^ (%)	49.0		51.0
Vascular dementia, 7 studies				
	N (cases)	6000 (151)	9034 (225)	8275 (181)
Model 2^a^	HR (95% CI)	1.11 (0.88‐1.39)	1.00	1.21 (0.98‐1.49)
	*I* ^2^ (%)	0		0
Model 3^b^	HR (95% CI)	1.09 (0.87‐1.36)	1.00	1.13 (0.92‐1.40)
	*I* ^2^ (%)	0		0

a Hazard ratio adjusted for age, smoking, education/socio‐economic status.

b Hazard ratios adjusted for age, smoking, education/socio‐economic status, diabetes, systolic blood pressure, total cholesterol, blood pressure lowering medication, cholesterol lowering medication, and glucose lowering medication.

As with all‐cause dementia, greater than or equal to 0.5% annual weight loss was associated with higher nonvascular dementia risk (1.41 [1.19‐1.67]), whereas greater than or equal to 0.5% annual weight gain was not associated with risk (0.99 [0.83‐1.19]; Table 2). For vascular dementia, opposing trends were observed: a trend towards increased risk was observed in those with greater than or equal to 0.5% annual weight gain (1.21 [0.98‐1.49]) but only in those with baseline mean age younger than 60 years (Figure S19). Weight loss was not associated with increased vascular dementia risk (Table 2).

## 4 DISCUSSION

This is the largest study to characterize the relationship between measures of body size and weight change with dementia outcomes. We demonstrated that the relationship between body size, weight change, and subsequent risk varies by dementia subtype. When considering all‐cause dementia risk, and hence its major subtype of nonvascular dementia, there was no evidence that excess body weight (measured by either BMI or WC) conferred higher risk. Rather, levels of BMI greater than or equal to 22.5 kg/m^2^ (and higher WCs) were associated with a slightly lower dementia risk in later life. Conversely, and in agreement with previous findings,^6^, ^7^, ^14^ individuals who were categorized as underweight had higher all‐cause dementia risk compared with those with lower‐normal BMI. For vascular dementia, only the highest levels of BMI and WC were associated with increased risk relative to lower‐normal BMI and the lowest fifth of WC.

The main novel finding, however, relates to how weight change appears to influence dementia risk in later life. Relative to weight maintenance, weight loss over follow‐up was associated with approximately 30% and 40% increased risk of all‐cause dementia and nonvascular dementia, respectively. The association between weight loss and nonvascular dementia may reflect the subclinical expression of prodromal dementia (ie, where an individual has early cognitive impairment but remains functionally independent), or in epidemiological terms, reverse causality. In support of this argument, weight loss has been observed in the preclinical stage of autosomal dominant Alzheimer disease, suggesting that decreasing BMI could be a consequence, rather than a risk factor, of dementia.^43^

An additional, albeit more speculative, explanation may be pathophysiological such as weight loss–induced cortical thinning, as cerebral atrophy is a characteristic of dementia. In a cohort of healthy elderly individuals, faster cognitive decline and accelerated atrophy rate were observed in those with relative weight loss greater than or equal to 5% (equivalent to greater than or equal to 1.2% annual loss) compared with those with relative weight loss less than 5% (equivalent to less than 1.2% annual loss).^44^ Similarly, a Norwegian study that assessed percent change in BMI in midlife reported that while greater than or equal to 5% loss (equivalent to approximately greater than or equal to 0.6% annual loss) was associated with increased risk of dementia‐related mortality, a gain of greater than or equal to 20% (equivalent to approximately greater than or equal to 2.2% annual gain) was associated with reduced risk.^45^

Conversely, for vascular dementia, weight gain was associated with a modest 20% increased risk but only in those aged younger than 60 years at study baseline. This is consistent not only with what we know about weight gain being a risk factor for other vascular conditions such as coronary heart disease^46^, ^47^ but also with the diminution of the strength in the association between vascular risk factors such as diabetes and blood pressure with vascular risk at older ages.^48^ Moreover, data from animal studies have indicated that weight gain is associated with increased vascular dementia risk.^49^

For all‐cause dementia, and its major subtype, that of nonvascular dementia, there was no evidence that carrying excess body weight (either in general or more centrally) conferred increased risk. Rather, individuals with a BMI of greater than or equal to 22.5 kg/m^2^ (and higher WCs) had a slightly lower risk of dementia in later life. Conversely, and in agreement with some previous findings,^6^, ^7^, ^14^ individuals who were categorized as underweight had a one‐quarter greater risk of developing all‐cause dementia compared with those with lower‐normal BMI. When we attempted to exclude those individuals who may have had undetected signs of cognitive impairment at study baseline by excluding the first few years of follow‐up, the relationship remained.

To date, evidence from Mendelian randomization studies has provided little support of a relationship between low levels of BMI with future risk of Alzheimer disease, implying that reverse causality or residual confounding may be driving the observed effect in observational studies. However, as Mendelian randomization studies do not use methods that are suited to capturing non‐linear associations, the potential for other explanations, other than reverse causality, to explain the observed association remains.^50^

For vascular dementia, the relationship with body size was similar to other vascular conditions with both underweight and obesity conferring increased risk. Underweight individuals had 75% greater vascular dementia risk, which was unaltered by excluding the first 5 years of data. Individuals with obesity had 50% greater risk, possibly due to the adverse effects of high levels of BMI on other vascular risk factors as the relationship was significantly attenuated after adjustment for vascular risk factors. An increased vascular dementia risk was observed for WC but only among those with the highest levels of central obesity.

Recent studies have suggested that the association between BMI and dementia risk is dependent on the age when BMI was assessed.^37^, ^51^ We investigated the association separately for midlife and late life by stratifying studies by their baseline mean age. Aside from the effect of weight gain on vascular dementia risk, our results indicated that there was no evidence of any difference between the two age groups, although the crude dichotomization of age precludes us from definitely concluding that there is no evidence of an age effect in the association between body size and dementia risk. Similarly, this study found no evidence to suggest that the associations reported herein differed between women and men. In addition to its large sample size and number of dementia cases, key strengths of the study included the ability to look at the effect of a measure of central obesity and the influence of weight change on the association between body size and dementia outcomes. BMI and WC were divided into five categories to allow the study of the relationship with dementia in more detail. The lowest fifth of WC was chosen as referent as four studies already included women with abdominal obesity in this group. Nevertheless, using the second or third fifth as referent would not have changed the relationship between WC and dementia. Limitations included the between‐study differences in design and methodologies used in the ascertainment of dementia as well as different lengths of study follow‐up. In regard to the latter, we attempted to address the potential for reverse causality by excluding the first 3, 5, or 10 years of follow‐up (depending on the data set). However, given the often long lag period between prodromal dementia until dementia onset, this may not have been a sufficiently long enough period of time to fully exclude the potential for reverse causality. Previous reports have indicated that it is necessary to exclude up to 20 years of follow‐up in order to fully negate the effects of inadvertently including individuals with early cognitive impairment at study baseline.^11^ In our analysis of weight change, we were unable to distinguish between intentional (eg, because of a diagnosis of hypertension or diabetes in midlife) versus unintentional weight loss (ie, because of pre‐existing disease), which may have diluted the observed associations. Studies that contributed to the weight change analysis also varied in the length of time between first and last weight measurement. We standardized weight change across studies by calculating annual percent weight change; however, this method assumes a consistent weight change over time, which may not be valid. In addition, we attempted to distinguish between vascular dementia and nonvascular dementia separately, but the dichotomization is problematic as the two subtypes frequently co‐occur.^52^ Finally, as body size (both underweight and overweight) is related to a wide range of chronic illnesses, and dementia is mainly a disease of aging, individuals may have died before they had the opportunity to develop dementia. We were unable to apply competing risks methodology in the current analysis, which may have resulted in an underestimation of the relationship between body size and dementia risk. It potentially could also explain why above normal levels of BMI and WC were not associated with increased all‐cause dementia risk.

Excess body weight was not associated with risk of all‐cause dementia and its major subtype of nonvascular dementia, whereas it was positively associated with risk of vascular dementia. Underweight was related to increased risk of all‐cause dementia and both its subtypes, possibly due to reverse causality. Weight loss in midlife to late life was associated with an increased risk of developing dementia and nonvascular dementia. Future studies should focus on examining the basis between weight loss and increased nonvascular dementia risk to determine if it has a pathophysiological basis or is due to limitations in the epidemiology. Given the known adverse effects of excess body weight on a wide range of health outcomes, from a public health perspective, maintaining a healthy body weight and minimizing weight fluctuation in adult life should continue to be widely promoted.

## CONFLICT OF INTEREST

MW has received personal fees from Amgen and Kirin outside the submitted work; JC has received grants from Idorsia outside the submitted work; EDA has received grants from NHS Blood and Transplant, British Heart Foundation, UK Medical Research Council, and National Institute for Health Research outside the submitted work; JK has received grants from Academy of Finland during the conduct of the study; MK has received grants from the Medical Research Council (MR/R024227/1), NIH National Institute on Aging (R01AG056477), Academy of Finland (311492), and Helsinki Institute of Life Sciences during the conduct of the study; EBL has received grants from NIH during the conduct of the study and personal fees from Up to Date outside the submitted work; ESL reported grants from Merck Inc outside the submitted work; NQ reported other from pharmaceutical industry outside the submitted work; EV reported grants from The Academy of Finland during the conduct of the study; KY serves on DSMBs for Takeda and Eli Lily outside the submitted work and is a member of the Beeson Scholars in Aging Scientific Advisory Board and of the Senate of the German Center for Neurodegenerative Diseases.

## Supporting information

Table S1: Study characteristics of male participants
Table S2: Study characteristics of female participants
Table S3: Adjusted hazard ratios with 95% confidence intervals (CI) of dementia by body mass index categories before and after left censoring of data
Table S4: Adjusted hazard ratios with 95% confidence intervals (CI) of dementia by body mass index categories for studies with all covariates listed in model 3
Table S5: Age, smoking, and education or socioeconomic status adjusted hazard ratios with 95% confidence intervals (CI) of all‐cause dementia by body mass index categories and subgroup
Table S6: Adjusted hazard ratios* with 95% confidence intervals of dementia by fifths of waist circumference for studies with all covariates listed in model 3
Fig. S1: Flow diagram
Fig. S2: Comparison of the association between body mass index (BMI) at study baseline with fatal and non‐fatal dementia and its major subtypes during follow‐up between non‐left censored and longest available left censored data (by 3, 5, or 10 years)
Fig. S3: Random effects pooled age adjusted hazard ratios with 95% confidence intervals of all‐cause dementia for body mass index <18.5 kg/m^2^ with body mass index 18.5–22.4 kg/m^2^ as referent
Fig. S4: Random effects pooled age adjusted hazard ratios with 95% confidence intervals of all‐cause dementia for body mass index 22.5–24.9 kg/m^2^ with body mass index 18.5–22.4 kg/m^2^ as referent.
Fig. S5: Random effects pooled age adjusted hazard ratios with 95% confidence intervals of all‐cause dementia for body mass index 25.0–29.9 kg/m^2^ with body mass index 18.5–22.4 kg/m^2^ as referent
Fig. S6: Random effects pooled age adjusted hazard ratios with 95% confidence intervals of all‐cause dementia for body mass index ≥30.0 kg/m^2^ with body mass index 18.5–22.4 kg/m^2^ as referent
Fig. S7: Associations between all‐cause dementia and body mass index by dementia ascertainment
Fig. S8: Associations between dementia and body mass index by study baseline mean age
Fig. S9: Associations between dementia and body mass index by study design
Fig. S10: Random effects pooled age adjusted hazard ratios with 95% confidence intervals of all‐cause dementia for second fifth of waist circumference with the first fifth of waist circumference as referent
Fig. S11: Random effects pooled age adjusted hazard ratios with 95% confidence intervals of all‐cause dementia for third fifth of waist circumference with the first fifth of waist circumference as referent
Fig. S12: Random effects pooled age adjusted hazard ratios with 95% confidence intervals of all‐cause dementia for fourth fifth of waist circumference with the first fifth of waist circumference as referent
Fig. S13: Random effects pooled age adjusted hazard ratios with 95% confidence intervals of all‐cause dementia for last fifth of waist circumference with the first fifth of waist circumference as referent
Fig. S14: Associations between all‐cause dementia and waist circumference by dementia ascertainment
Fig. S15: Associations between dementia and waist circumference by study design
Fig. S16: Associations between dementia and waist circumference by study baseline mean age
Fig. S17: Random effects pooled age adjusted hazard ratios with 95% confidence intervals of all‐cause dementia for ≥0.5% weight loss per year with <0.5% weight change has referent
Fig. S18: Random effects pooled age adjusted hazard ratios with 95% confidence intervals of all‐cause dementia for ≥0.5% weight gain per year with <0.5% weight change has referent
Fig. S19: Associations between all‐cause dementia and annual percent weight change by study baseline mean age
Fig. S20: Associations between all‐cause dementia and annual percent weight change by dementia ascertainment
