# Supplementary material for: Association of anthropometry and weight change with risk of dementia and its major subtypes: A meta‐analysis consisting 2.8 million adults with 57 294 cases of dementia
Source: Obes Rev. 2020 Jan 3;21(4):e12989. doi: 10.1111/obr.12989 (PMC7079047; doi:10.1111/obr.12989)
Supplement: Supplementary file 1 — Table S1: Study characteristics of male participants Table S2: Study characteristics of female participants Table S3: Adjusted hazard ratios with 95% confidence intervals (CI) of dementia by body mass index categories before and after left censoring of data Table S4: Adjusted hazard ratios with 95% confidence intervals (CI) of dementia by body mass index categories for studies with all covariates listed in model 3 Table S5: Age, smoking, and education or socioeconomic status adjusted hazard ratios with 95% confidence intervals (CI) of all‐cause dementia by body mass index categories and subgroup Table S6: Adjusted hazard ratios* with 95% confidence intervals of dementia by fifths of waist circumference for studies with all covariates listed in model 3 Fig. S1: Flow diagram Fig. S2: Comparison of the association between body mass index (BMI) at study baseline with fatal and non‐fatal dementia and its major subtypes during follow‐up between non‐left censored and longest available left censored data (by 3, 5, or 10 years) Fig. S3: Random effects pooled age adjusted hazard ratios with 95% confidence intervals of all‐cause dementia for body mass index <18.5 kg/m2 with body mass index 18.5–22.4 kg/m2 as referent Fig. S4: Random effects pooled age adjusted hazard ratios with 95% confidence intervals of all‐cause dementia for body mass index 22.5–24.9 kg/m2 with body mass index 18.5–22.4 kg/m2 as referent. Fig. S5: Random effects pooled age adjusted hazard ratios with 95% confidence intervals of all‐cause dementia for body mass index 25.0–29.9 kg/m2 with body mass index 18.5–22.4 kg/m2 as referent Fig. S6: Random effects pooled age adjusted hazard ratios with 95% confidence intervals of all‐cause dementia for body mass index ≥30.0 kg/m2 with body mass index 18.5–22.4 kg/m2 as referent Fig. S7: Associations between all‐cause dementia and body mass index by dementia ascertainment Fig. S8: Associations between dementia and body mass index by study baseline mean age Fig. S9: As [file OBR-21-e12989-s001.docx]

**Association of anthropometry and weight change with risk of dementia and its major subtypes: a meta-analysis consisting 2.8 million adults with 57,294 cases of dementia**

Crystal Man Ying Lee, Mark Woodward, G. David Batty, Alexa S Beiser, Steven Bell, Claudine Berr, Espen Bjertness, John Chalmers, Robert Clarke, Jean-Francois Dartigues, Kendra Davis-Plourde, Stéphanie Debette, Emanuele Di Angelantonio, Catherine Feart, Ruth Frikke-Schmidt, John Gregson, Mary N. Haan, Linda B. Hassing, Kathleen M. Hayden, Marieke P. Hoevenaar-Blom, Jaakko Kaprio, Mika Kivimaki, Georgios Lappas, Eric B. Larson, Erin S. LeBlanc, Anne Lee, Li-Yung Lui, Eric P. Moll van Charante, Toshiharu Ninomiya, Liv Tybjærg Nordestgaard, Tomoyuki Ohara, Toshiaki Ohkuma, Teemu Palviainen, Karine Peres, Ruth Peters, Nawab Qizilbash, Edo Richard, Annika Rosengren, Sudha Seshadri, Martin Shipley, Archana Singh-Manoux, Bjorn Heine Strand, Willem A. van Gool, Eero Vuoksimaa, Kristine Yaffe, Rachel R. Huxley

**Table S1: Study characteristics of male participants**

| **Study** | **N** | **Mean follow-up**  **(year)** | **Dementia cases** | | | **Mean (SD) at study baseline** | | | | **% at study baseline** | |
| --- | --- | --- | --- | --- | --- | --- | --- | --- | --- | --- | --- |
|  |  |  | **All-cause** | **Vascular** | **Non-vascular** | **Age**  **(year)** | **BMI (kg/m^2^)** | **WC**  **(cm)** | **SBP**  **(mmHg)** | **Prevalent diabetes** | **Current smoker** |
| ACT | 1811 | 8.0 | 412 | 48 | 364 | 73.4 (5.8) | 27.5 (4.1) | 100.7 (11.1) | 138.1 (19.3) | 11.8 | 4.1 |
| ADVANCE | 6404 | 4.8 | 58 | - | - | 66.0 (6.0) | 28.0 (4.7) | 100.7 (12.7) | 145.0 (21.0) | 100 | 17.0 |
| AMI | 345 | 5.6 | 42 | 4 | 38 | 74.8 (6.0) | 28.2 (4.1) | 103.1 (11.6) | 151.8 (21.2) | 13.3 | 5.5 |
| CCHS | 4006 | 16.1 | 339 | 54 | 316 | 56.9 (15.1) | 26.1 (3.9) | 94.8 (11.6) | 140.8 (21.1) | 5.9 | 52.6 |
| CCMS | 1358 | 4.0 | 211 | 37 | 174 | 73.3 (6.1) | 26.6 (3.8) | - | - | 11.8 | 4.3 |
| CGPS | 46795 | 7.6 | 854 | 100 | 797 | 58.4 (13.1) | 26.8 (3.7) | 96.6 (11.0) | 144.7 (20.0) | 4.9 | 18.2 |
| CPRD | 885706 | 9.5 | 17026 | - | - | 56.3 (12.2) | 27.4 (4.7) | - | - | 8.1 | 26.3 |
| FHS | 981 | 7.7 | 99 | 18 | 81 | 71.5 (7.3) | 28.2 (4.3) | 102.6 (10.9) | 135.0 (19.5) | 18.5 | 65.7 |
| FTC | 12630 | 31.3 | 303 | - | - | 35.6 (13.5) | 23.9 (3.1) | - | - | 1.3 | 42.2 |
| GPO | 873 | 27.5 | 6 | - | - | 48.6 (7.9) | 25.0 (3.2) | - | 135.2 (21.3) | 1.3 | 58.7 |
| Hisayama Study | 497 | 11.1 | 116 | 57 | 59 | 70.0 (7.0) | 21.9 (2.9) | 80.2  (8.6) | 139.0 (23.0) | 14.7 | 45.7 |
| HSE & SHS | 41092 | 9.0 | 201 | - | - | 47.7 (17.9) | 26.8 (4.2) | 95.6 (11.9) | 134.5 (17.1) | 3.9 | 22.8 |
| HYVET | 1321 | 2.2 | 105 | 32 | 73 | 83.3 (3.0) | 24.7 (3.2) | - | 172.0 (8.0) | 9.9 | 12.5 |
| NCS | 20262 | 24.7 | 515 | - | - | 51.2 (3.9) | 25.1 (3.0) | - | 137.3 (16.0) | 0.7 | 52.3 |
| OCTO-Twin | 357 | 35.9 | 82 | 27 | 55 | 52.1 (4.2) | 24.7 (2.3) | - | - | 17.9 | 70.0 |
| preDIVA | 1607 | 6.0 | 100 | 8 | 82 | 74.3 (2.5) | 27.0 (3.5) | 102.0 (9.9) | 156.5 (21.3) | 19.4 | 13.8 |
| Preventiva | 7394 | 26.8 | 788 | 173 | 615 | 51.6 (2.3) | 25.5 (3.3) | - | 148.7 (21.9) | 2.0 | 49.2 |
| PROGRESS | 4121 | 3.9 | 263 | - | - | 64.0 (9.0) | 25.7 (3.5) | - | 146.0 (19.0) | 13.0 | 24.0 |
| 3C | 2595 | 8.7 | 292 | 67 | 225 | 73.2 (4.9) | 26.2 (3.4) | 95.9 (10.1) | 150.7 (21.5) | 12.3 | 8.1 |
| UK Biobank | 220773 | 6.9 | 202 | - | - | 57.3 (8.2) | 27.8 (4.2) | 96.9 (11.3) | 140.7 (17.4) | 6.9 | 12.3 |
| Whitehall I | 17167 | 26.9 | 288 | - | - | 52.1 (6.8) | 24.8 (3.0) | - | 136.6 (21.3) | 1.3 | 41.8 |
| Whitehall II | 3620 | 16.3 | 97 | 20 | 24 | 55.5 (6.0) | 26.0 (3.5) | 91.9 (10.0) | 123.5 (16.1) | 5.2 | 9.2 |

ACT = Adult Changes in Thought study; ADVANCE = Action in Diabetes and Vascular Disease Preteraz and Diamicron MR Controlled Evaluation trial; AMI = Aging Multidisciplinary Investigation cohort; CCHS = The Copenhagen City Heart Study ; CCMS = Cache County Memory Study; CGPS = The Copenhagen General Population Study; CPRD = Clinical Practice Research Datalink; FHS = Framingham Heart Study; FTC = Finnish Twin Cohort; GPO = General Post Office Study; HSE & SHS = Health Survey for England and Sottish Health Survey; HYVET = Hypertension in the Very Elderly Trial; NCS = Norwegian Counties Study; OCTO Twin = Origins of Variance in the Old-Old; preDIVA = Prevention of Dementia by Intensive Vascular Care trial; Preventiva = Primary Prevention Study; PROGRESS = The Perindopril Protection Against Recurrent Stroke Study; 3C = Three City study; SD = standard deviation; BMI = body mass index; WC = waist circumference; SBP = systolic blood pressure;

**Table S2: Study characteristics of female participants**

| **Study** | **N** | **Mean follow-up**  **(year)** | **Dementia cases** | | | **Mean (SD) at study baseline** | | | | **% at study baseline** | |
| --- | --- | --- | --- | --- | --- | --- | --- | --- | --- | --- | --- |
|  |  |  | **All-cause** | **Vascular** | **Non-vascular** | **Age**  **(year)** | **BMI**  **(kg/m^2^)** | **WC**  **(cm)** | **SBP**  **(mmHg)** | **Prevalent diabetes** | **Current smoker** |
| ACT | 2532 | 8.6 | 684 | 62 | 622 | 74.6 (6.5) | 27.3 (5.4) | 90.3 (13.9) | 141.0 (21.4) | 9.6 | 5.5 |
| ADVANCE | 4732 | 4.8 | 51 | - | - | 66.0 (6.0) | 28.8 (5.7) | 95.6 (13.1) | 145.0 (22.0) | 100 | 9.0 |
| AMI | 218 | 5.8 | 23 | 3 | 20 | 74.8 (6.0) | 27.2 (4.9) | 95.3 (13.5) | 143.8 (19.7) | 10.6 | 1.8 |
| CCHS | 5031 | 17.7 | 630 | 64 | 604 | 58.5 (15.0) | 25.2 (4.6) | 82.4 (12.2) | 137.5 (23.5) | 2.9 | 46.1 |
| CCMS | 1827 | 4.0 | 296 | 38 | 258 | 74.4 (6.5) | 26.2 (4.9) | - | - | 9.5 | 0.9 |
| CGPS | 57711 | 7.7 | 1052 | 90 | 1004 | 57.6 (13.1) | 25.6 (4.6) | 84.6 (12.1) | 138.9 (22.1) | 2.8 | 16.4 |
| CPRD | 1072485 | 9.6 | 28481 | - | - | 56.5 (13.3) | 26.9 (5.7) | - | - | 5.6 | 21.2 |
| FHS | 1251 | 8.1 | 190 | 30 | 160 | 73.1 (7.6) | 27.2 (5.1) | 97.2 (14.0) | 136.2 (21.5) | 12.2 | 48.0 |
| FTC | 13184 | 32.8 | 657 | - | - | 36.9 (15.2) | 22.7 (3.6) | - | - | 1.7 | 23.2 |
| GPO | 512 | 33.1 | 12 | - | - | 47.0 (6.9) | 24.8 (3.9) | - | 126.6 (20.9) | 0.6 | 50.2 |
| Hisayama study | 695 | 12.0 | 234 | 60 | 174 | 71.0 (7.0) | 22.4 (3.4) | 81.5 (10.7) | 141.0 (24.0) | 13.7 | 7.0 |
| HSE & SHS | 49593 | 9.3 | 323 | - | - | 47.6 (18.0) | 26.6 (5.3) | 84.7 (12.7) | 129.2 (20.4) | 2.7 | 22.3 |
| HYVET | 2016 | 2.3 | 158 | 52 | 106 | 83.6 (3.2) | 24.6 (3.9) | - | 173.6  (8.7) | 10.0 | 1.9 |
| NCS | 20716 | 27.1 | 658 | - | - | 51.2 (3.9) | 24.8 (4.1) | - | 133.0 (17.8) | 0.5 | 35.6 |
| OCTO-Twin | 795 | 37.9 | 230 | 49 | 181 | 52.7 (4.2) | 24.7 (3.2) | - | - | 17.0 | 30.5 |
| preDIVA | 1919 | 6.3 | 133 | 11 | 113 | 74.4 (2.4) | 27.8 (4.6) | 97.5 (12.2) | 154.4 (21.4) | 17.4 | 12.9 |
| PROGRESS | 1744 | 3.9 | 117 | - | - | 65.0 (10.0) | 25.7 (4.4) | - | 149.0 (20.0) | 11.0 | 11.0 |
| SOF | 1019 | 19.9 | 232 | 28 | 204 | 68.2 (2.8) | 26.7 (4.4) | 82.6 (10.6) | 138.4 (17.5) | 2.6 | 4.5 |
| 3C | 4126 | 9.3 | 540 | 101 | 439 | 73.4 (4.8) | 25.4 (4.3) | 84.1 (11.4) | 142.8 (20.6) | 6.9 | 3.7 |
| UK Biobank | 265502 | 7.0 | 142 | - | - | 56.9 (8.0) | 27.1 (5.2) | 84.6 (12.5) | 135.1 (19.2) | 3.4 | 8.8 |
| Whitehall II | 1430 | 16.5 | 52 | 8 | 18 | 55.9 (6.0) | 26.3 (4.9) | 80.7 (11.8) | 121.6 (17.3) | 6.0 | 11.7 |

ACT = Adult Changes in Thought study; ADVANCE = Action in Diabetes and Vascular Disease Preteraz and Diamicron MR Controlled Evaluation trial; AMI = Aging Multidisciplinary Investigation cohort; CCHS = The Copenhagen City Heart Study ; CCMS = Cache County Memory Study; CGPS = The Copenhagen General Population Study; CPRD = Clinical Practice Research Datalink; FHS = Framingham Heart Study; FTC = Finnish Twin Cohort; GPO = General Post Office Study; HSE & SHS = Health Survey for England and Sottish Health Survey; HYVET = Hypertension in the Very Elderly Trial; NCS = Norwegian Counties Study; OCTO Twin = Origins of Variance in the Old-Old; preDIVA = Prevention of Dementia by Intensive Vascular Care trial; PROGRESS = The Perindopril Protection Against Recurrent Stroke Study; SOF = Study of Osteoporotic Fractures; 3C = Three City study; SD = standard deviation; BMI = body mass index; WC = waist circumference; SBP = systolic blood pressure;

**Table S3: Adjusted hazard ratios with 95% confidence intervals (CI) of dementia by body mass index categories before and after left censoring of data**

| **Dementia type** | | **Body mass index (kg/m^2^)** | | | | | |
| --- | --- | --- | --- | --- | --- | --- | --- |
|  |  | **<18.5** | **18.5–22.4** | **22.5–24.9** | **25.0–29.9** | **≥30.0** |  |
| **All-cause dementia – 19 studies** | | | | | | | |
| **Non-left censored** | | | | | | | |
| Model 2* | Hazard ratio (95% CI) | 1.26 (1.20, 1.31) | 1.00 | 0.85 (0.83, 0.87) | 0.83 (0.78, 0.89) | 0.86 (0.78, 0.95) |  |
|  | I^2^ (%) | 0 |  | 0 | 60.7 | 75.9 |  |
| Model 3^#^ | Hazard ratio (95% CI) | 1.27 (1.21, 1.33) | 1.00 | 0.85 (0.83, 0.87) | 0.81 (0.76, 0.87) | 0.82 (0.74, 0.91) |  |
|  | I^2^ (%) | 0 |  | 0 | 59.0 | 75.1 |  |
| **Left censored (2 studies left censored by 3 years; 7 studies left censored by 5 years; 10 study left censored by 10 years)** | | | | | | | |
| Model 2* | Hazard ratio (95% CI) | 1.35 (1.24, 1.46) | 1.00 | 0.90 (0.84, 0.98) | 0.88 (0.77, 1.01) | 0.92 (0.76, 1.12) |  |
|  | I^2^ (%) | 0 |  | 21.4 | 72.2 | 78.5 |  |
| Model 3^#^ | Hazard ratio (95% CI) | 1.36 (1.25, 1.48) | 1.00 | 0.90 (0.83, 0.97) | 0.86 (0.76, 0.99) | 0.86 (0.71, 1.04) |  |
|  | I^2^ (%) | 0 |  | 23.4 | 72.1 | 76.5 |  |
| **Non-vascular dementia – 10 studies** | | | | | | | |
| **Non-left censored** | | | | | | | |
| Model 2* | Hazard ratio (95% CI) | 1.28 (1.05, 1.56) | 1.00 | 0.88 (0.81, 0.96) | 0.81 (0.75, 0.88) | 0.83 (0.72, 0.96) |  |
|  | I^2^ (%) | 0 |  | 0 | 2.2 | 41.3 |  |
| Model 3^#^ | Hazard ratio (95% CI) | 1.27 (1.04, 1.54) | 1.00 | 0.87 (0.80, 0.95) | 0.80 (0.74, 0.87) | 0.80 (0.69, 0.94) |  |
|  | I^2^ (%) | 0 |  | 0 | 7.6 | 42.8 |  |
| **Left censored (5 studies left censored by 5 years; 5 studies left censored by 10 years)** | | | | | | | |
| Model 2* | Hazard ratio (95% CI) | 1.28 (0.95, 1.74) | 1.00 | 0.91 (0.79, 1.04) | 0.90 (0.79, 1.02) | 0.96 (0.82, 1.12) |  |
|  | I^2^ (%) | 0 |  | 0 | 0 | 0 |  |
| Model 3^#^ | Hazard ratio (95% CI) | 1.26 (0.93, 1.72) | 1.00 | 0.91 (0.79, 1.04) | 0.89 (0.78, 1.01) | 0.91 (0.78, 1.08) |  |
|  | I^2^ (%) | 0 |  | 0 | 0 | 0 |  |
| **Vascular dementia – 8 studies** | | | | | | | |
| **Non-left censored** | | | | | | | |
| Model 2* | Hazard ratio (95% CI) | 1.88 (1.07, 3.32) | 1.00 | 1.05 (0.81, 1.36) | 1.08 (0.81, 1.43) | 1.45 (1.00, 2.09) |  |
|  | I^2^ (%) | 32.0 |  | 19.6 | 35.3 | 41.4 |  |
| Model 3^#^ | Hazard ratio (95% CI) | 2.11 (1.17, 3.83) | 1.00 | 1.02 (0.78, 1.34) | 0.98 (0.73, 1.30) | 1.18 (0.84, 1.68) |  |
|  | I^2^ (%) | 36.3 |  | 22.7 | 36.6 | 32.2 |  |
| **Left censored (4 studies left censored by 5 years; 4 studies left censored by 10 years)** | | | | | | | |
| Model 2* | Hazard ratio (95% CI) | - | 1.00 | 1.15 (0.85, 1.57) | 1.20 (0.89, 1.61) | 1.59 (1.02, 2.48) |  |
|  | I^2^ (%) |  |  | 0 | 0 | 26.2 |  |
| Model 3^#^ | Hazard ratio (95% CI) | - | 1.00 | 1.10 (0.79, 1.52) | 1.06 (0.78, 1.43) | 1.27 (0.79, 2.06) |  |
|  | I^2^ (%) |  |  | 5.7 | 0 | 33.3 |  |

*Hazard ratio adjusted for age, smoking, education/socioeconomic status

^#^Hazard ratios adjusted for age, smoking, education/socioeconomic status, diabetes, systolic blood pressure, total cholesterol, blood pressure lowering medication, cholesterol lowering medication, and glucose lowering medication

**Table S4: Adjusted hazard ratios with 95% confidence intervals (CI) of dementia by body mass index categories for studies with all covariates listed in model 3**

| **Dementia type** |  | **Body mass index (kg/m^2^)** | | | | | | |
| --- | --- | --- | --- | --- | --- | --- | --- | --- |
|  |  | **<18.5** | **18.5–22.4** | **22.5–24.9** | **25.0–29.9** | | **≥30.0** | |
| **All-cause dementia – 10 studies** | N (cases) | 1353 (94) | 22569 (1005) | 37044 (1408) | 60170 (2406) | 26131 (998) | |  |
| Model 2* | Hazard ratio (95% CI) | 1.35 (1.09, 1.68) | 1.00 | 0.89 (0.82, 0.96) | 0.84 (0.77, 0.90) | | 0.90 (0.77, 1.06) | |
|  | I^2^ (%) | 0 |  | 0 | 0 | | 53.8 | |
| Model 3^#^ | Hazard ratio (95% CI) | 1.35 (1.09, 1.68) | 1.00 | 0.88 (0.81, 0.96) | 0.83 (0.76, 0.90) | | 0.86 (0.72, 1.03) | |
|  | I^2^ (%) | 0 |  | 0 | 6.3 | | 58.6 | |
| **Non-vascular dementia – 9 studies** | N (cases) | 1353 (77) | 24589 (890) | 35029 (1233) | 55650 (2062) | 22602 (824) | |  |
| Model 2* | Hazard ratio (95% CI) | 1.26 (1.00, 1.60) | 1.00 | 0.88 (0.81, 0.96) | 0.83 (0.75, 0.91) | 0.83 (0.71, 0.98) | |  |
|  | I^2^ (%) | 0 |  | 0 | 16.4 | 46.8 | |  |
| Model 3^#^ | Hazard ratio (95% CI) | 1.26 (0.99, 1.59) | 1.00 | 0.89 (0.81, 0.97) | 0.82 (0.75, 0.90) | 0.82 (0.70, 0.96) | |  |
|  | I^2^ (%) | 0 |  | 0 | 8.8 | 42.3 | |  |
| **Vascular dementia – 8 studies** | N (cases) | 1199 (15) | 24163 (111) | 34235 (171) | 54009 (311) | 21892 (139) | |  |
| Model 2* | Hazard ratio (95% CI) | 2.43 (1.31, 4.51) | 1.00 | 0.94 (0.66, 1.34) | 1.01 (0.74, 1.37) | | 1.41 (1.00, 1.99) | |
|  | I^2^ (%) | 17.6 |  | 39.5 | 35.8 | | 29.7 | |
| Model 3^#^ | Hazard ratio (95% CI) | 2.54 (1.34, 4.83) | 1.00 | 0.91 (0.63, 1.30) | 0.95 (0.69. 1.31) | | 1.26 (0.87, 1.84) | |
|  | I^2^ (%) | 22.0 |  | 41.1 | 38.4 | | 44.3 | |

*Hazard ratio adjusted for age, smoking, education/socioeconomic status

^#^Hazard ratios adjusted for age, smoking, education/socioeconomic status, diabetes, systolic blood pressure, total cholesterol, blood pressure lowering medication, cholesterol lowering medication, and glucose lowering medication

**Table S5: Age, smoking, and education or socioeconomic status adjusted hazard ratios with 95% confidence intervals (CI) of all-cause dementia by body mass index categories and subgroup**

| **Subgroup** |  | **Body mass index (kg/m^2^)** | | | | |
| --- | --- | --- | --- | --- | --- | --- |
|  |  | **<18.5** | **18.5–22.4** | **22.5–24.9** | **25.0–29.9** | **≥30.0** |
| Overall | N (cases) | 44408 (2510) | 431624 (12110) | 583832 (13402) | 1067974 (20573) | 661475 (8675) |
|  | Hazard ratio (95% CI) | 1.26 (1.20, 1.31) | 1.00 | 0.85 (0.83, 0.88) | 0.86 (0.81, 0.92) | 0.91 (0.83, 1.01) |
|  | I^2^ (%) | 0 |  | 0 | 65.5 | 78.4 |
| Excluded Clinical Practice Research Datalink | N (cases) | 7094 (212) | 126461 (2154) | 187748 (3011) | 338627 (4765) | 171192 (1621) |
|  | Hazard ratio (95% CI) | 1.36 (1.17, 1.57) | 1.00 | 0.90 (0.85, 0.95) | 0.88 (0.81, 0.96) | 0.96 (0.84, 1.09) |
|  | I^2^ (%) | 0 |  | 0 | 47.7 | 66.2 |
| Restricted to measured height and weight | N (cases) | 43359 (2488) | 420386 (11789) | 576047 (12890) | 1060763 (19851) | 659836 (8495) |
|  | Hazard ratio (95% CI) | 1.26 (1.20, 1.32) | 1.00 | 0.85 (0.83, 0.87) | 0.83 (0.77, 0.88) | 0.85 (0.77, 0.94) |
|  | I^2^ (%) | 0 |  | 0 | 59.5 | 73.2 |
| Clinical trials | N (cases) | 194 (20) | 3125 (181) | 5155 (233) | 9922 (386) | 5325 (165) |
|  | Hazard ratio (95% CI) | 1.36 (0.84, 2.21) | 1.00 | 0.80 (0.65, 0.98) | 0.80 (0.66, 0.97) | 0.92 (0.62, 1.35) |
|  | I^2^ (%) | 0 |  | 0 | 0 | 60.2 |
| Non-trial studies | N (cases) | 44214 (2490) | 428499 (11929) | 578677 (13169) | 1058052 (20187) | 656150 (8510) |
|  | Hazard ratio (95% CI) | 1.26 (1.20, 1.31) | 1.00 | 0.87 (0.84, 0.90) | 0.87 (0.81, 0.94) | 0.91 (0.82, 1.01) |
|  | I^2^ (%) | 0 |  | 8.4 | 71.1 | 80.1 |

**Table S6: Adjusted hazard ratios* with 95% confidence intervals of dementia by fifths of waist circumference for studies with all covariates listed in model 3**

| **Dementia type** |  | **Waist circumference** | | | | | | |
| --- | --- | --- | --- | --- | --- | --- | --- | --- |
|  |  | **1^st^ fifth** | **2^nd^ fifth** | **3^rd^ fifth** | **4^th^ fifth** | **5^th^ fifth** | |  |
| **All-cause dementia – 9 studies** | N (cases) | 30985 (1029) | 29164 (1037) | 28279 (1148) | 29199 (1193) | 28757 (1211) |  |  |
| Model 2* | Hazard ratio (95% CI) | 1.00 | 0.88 (0.80, 0.95) | 0.89 (0.81, 0.98) | 0.83 (0.76, 0.90) | 0.87 (0.76, 1.00) | |  |
|  | I^2^ (%) |  | 0 | 14.2 | 0 | 46.7 | |  |
| Model 3^#^ | Hazard ratio (95% CI) | 1.00 | 0.87 (0.79, 0.95) | 0.86 (0.78, 0.96) | 0·80 (0.73, 0.87) | 0.83 (0.72, 0.96) | |  |
|  | I^2^ (%) |  | 0 | 21.5 | 0 | 49.7 | |  |
| **Non-vascular dementia – 8 studies** | N (cases) | 28694 (911) | 27001 (911) | 26182 (1009) | 26815 (1024) | 26558 (1034) |  |  |
| Model 2* | Hazard ratio (95% CI) | 1.00 | 0.87 (0.79, 0.95) | 0.87 (0.79, 0.96) | 0.79 (0.72, 0.86) | 0.82 (0.70, 0.96) | |  |
|  | I^2^ (%) |  | 0 | 9.6 | 0 | 50.7 | |  |
| Model 3^#^ | Hazard ratio (95% CI) | 1.00 | 0.86 (0.78, 0.94) | 0.85 (0.76, 0.94) | 0.77 (0.70, 0.84) | 0.79 (0.66, 0.93) | |  |
|  | I^2^ (%) |  | 0 | 14.2 | 0 | 57.0 | |  |
| **Vascular dementia – 7 studies** | N (cases) | 25845 (163) | 28036 (96) | 26321 (119) | 25483 (135) | 26115 (150) | |  |
| Model 2* | Hazard ratio (95% CI) | 1.00 | 1.08 (0.82, 1.43) | 1.10 (0.84, 1.45) | 1.13 (0.87, 1.47) | 1.30 (1.00, 1.69) | |  |
|  | I^2^ (%) |  | 0 | 0 | 0 | 0 | |  |
| Model 3^#^ | Hazard ratio (95% CI) | 1.00 | 1.05 (0.79, 1.38) | 1.04 (0.79, 1.36) | 1.03 (0.79, 1.35) | 1.10(0.84, 1.44) | |  |
|  | I^2^ (%) |  | 0 | 0 | 0 | 0 | |  |

*Hazard ratio adjusted for age, smoking, education/socioeconomic status

^#^Hazard ratios adjusted for age, smoking, education/socioeconomic status, diabetes, systolic blood pressure, total cholesterol, blood pressure lowering medication, cholesterol lowering medication, and glucose lowering medication

**Fig. S1: Flow diagram**


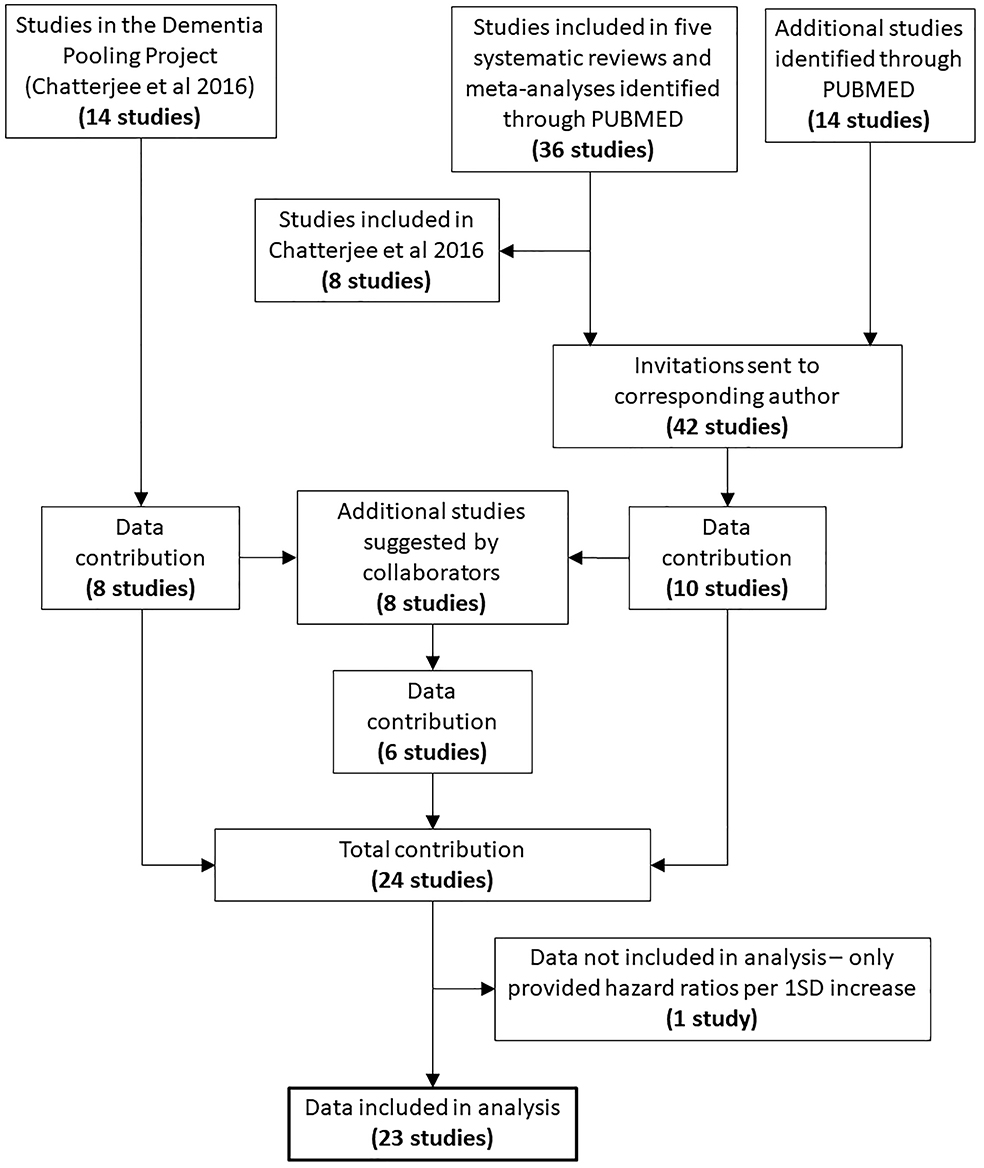


**Fig. S2: Comparison of the association between body mass index (BMI) at study baseline with fatal and non-fatal dementia and its major subtypes during follow-up between non-left censored and longest available left censored data (by 3, 5, or 10 years)**

**
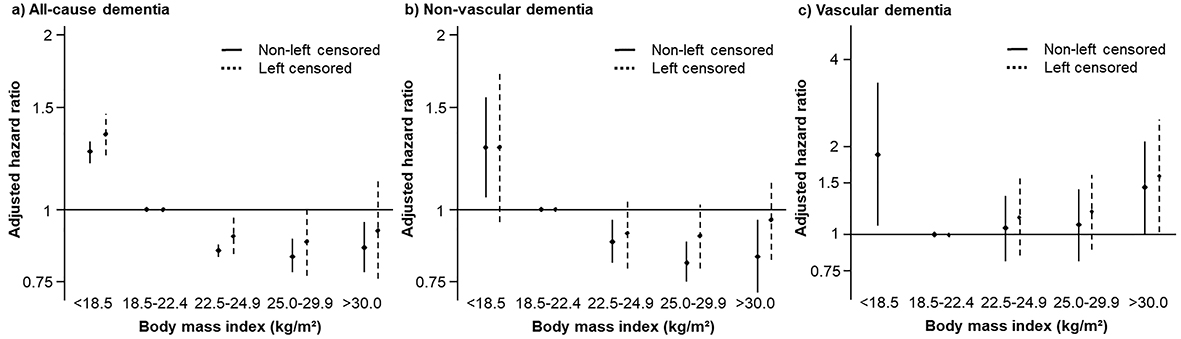
**

Hazard ratios and 95% confidence intervals adjusted for age, smoking, and education or socioeconomic status

**Fig. S3: Random effects pooled age adjusted hazard ratios with 95% confidence intervals of all-cause dementia for body mass index <18.5 kg/m^2^ with body mass index 18.5–22.4 kg/m^2^ as referent**

**Fig. S4: Random effects pooled age adjusted hazard ratios with 95% confidence intervals of all-cause dementia for body mass index 22.5–24.9 kg/m^2^ with body mass index 18.5–22.4 kg/m^2^ as referent.**

**Fig. S5: Random effects pooled age adjusted hazard ratios with 95% confidence intervals of all-cause dementia for body mass index 25.0–29.9 kg/m^2^ with body mass index 18.5–22.4 kg/m^2^ as referent**


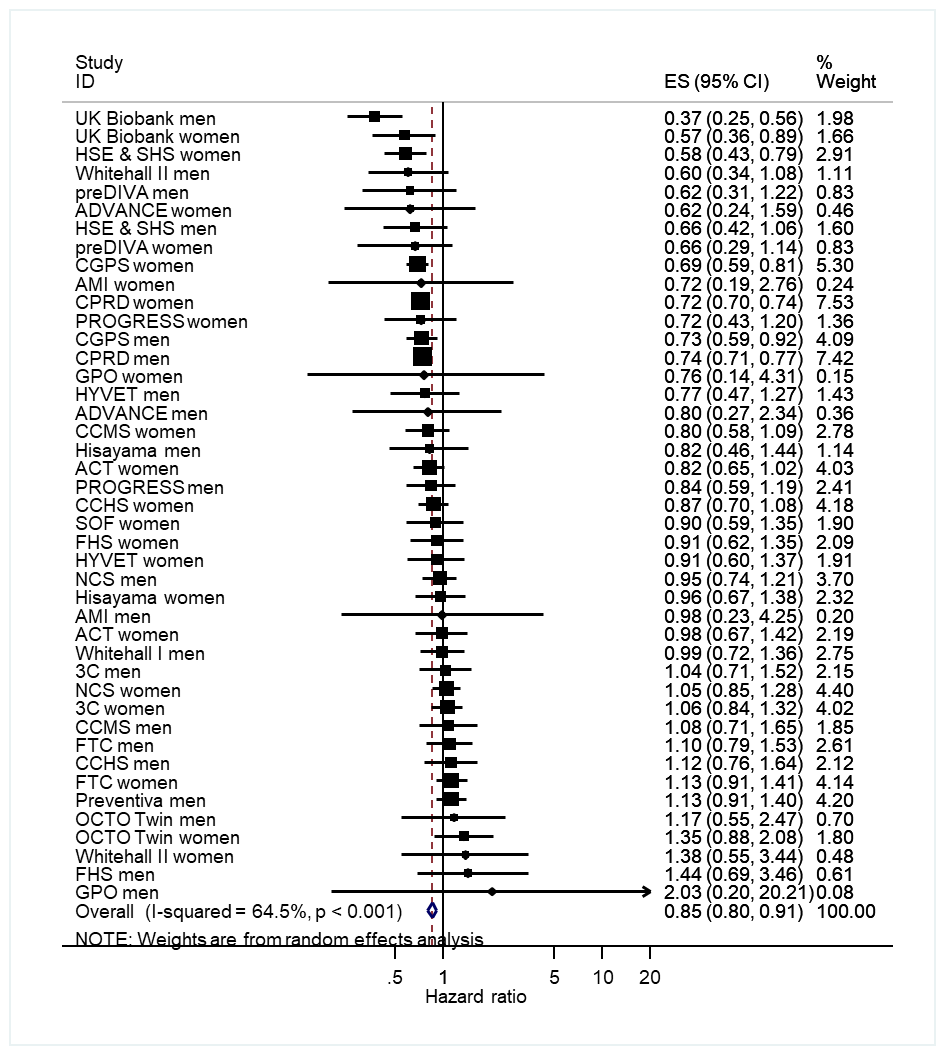


**Fig. S6: Random effects pooled age adjusted hazard ratios with 95% confidence intervals of all-cause dementia for body mass index ≥30.0 kg/m^2^ with body mass index 18.5–22.4 kg/m^2^ as referent**


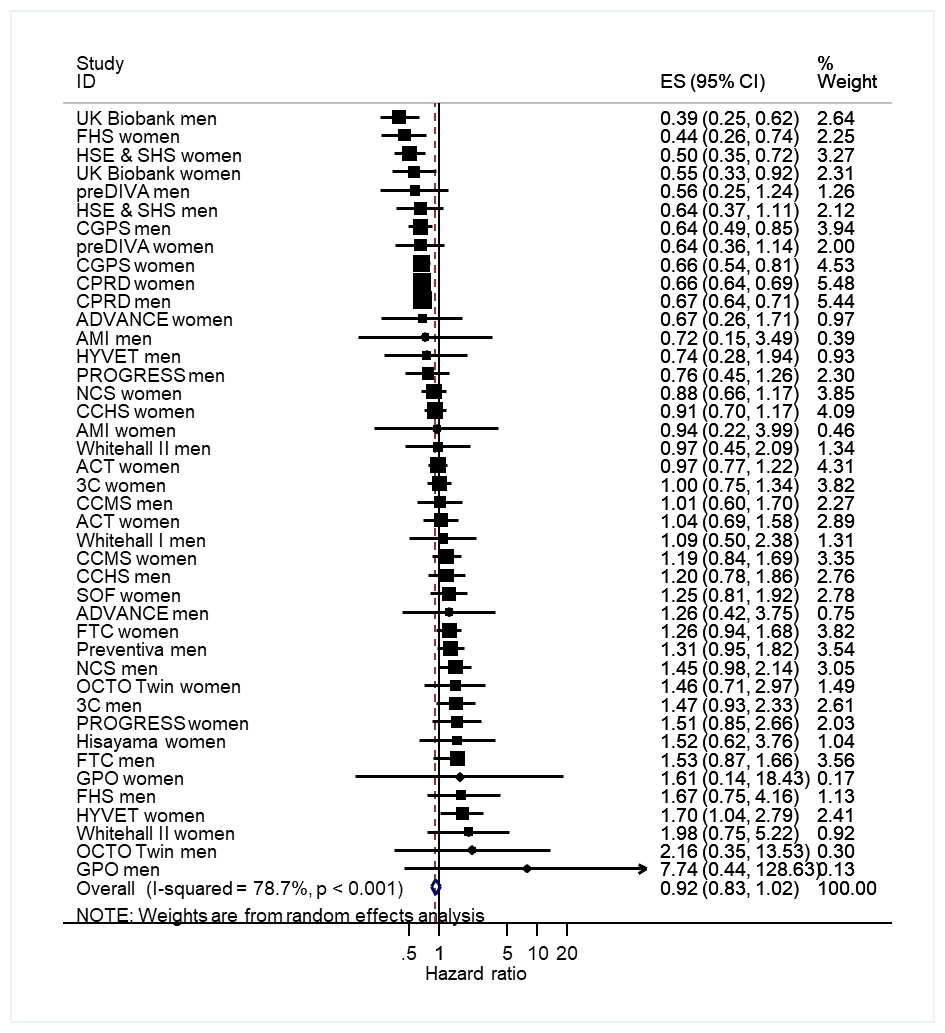


**Fig. S7: Associations between all-cause dementia and body mass index by dementia ascertainment**


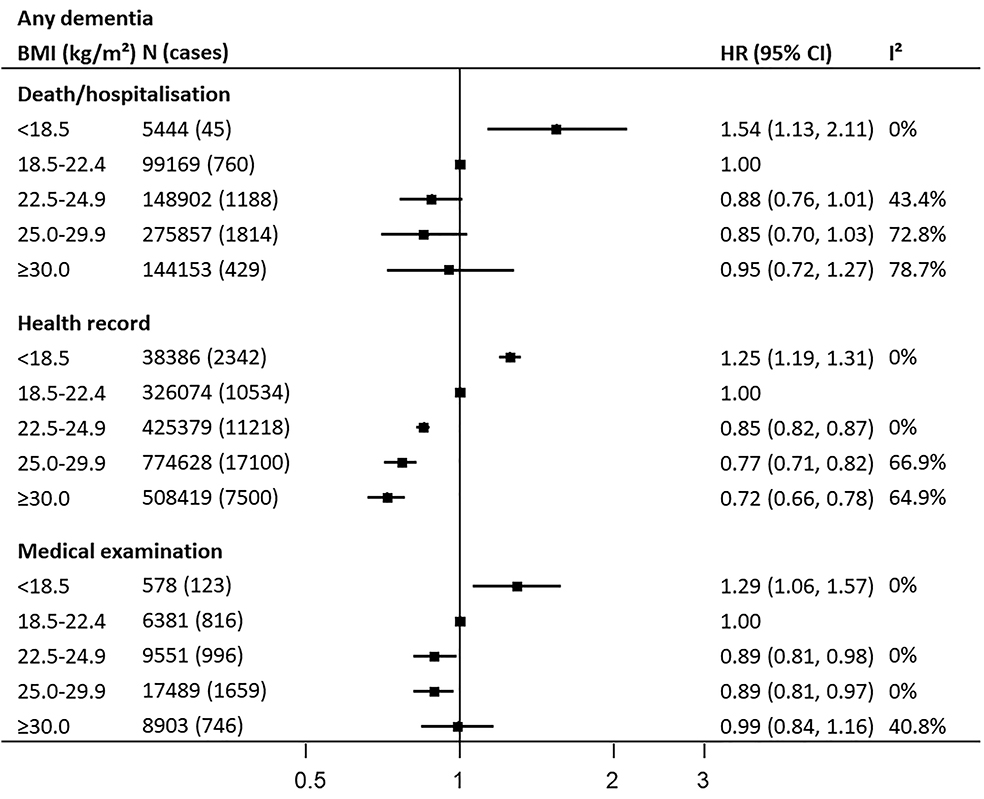


Hazard ratios adjusted for age, smoking, and education or socioeconomic status

**Fig. S8: Associations between dementia and body mass index by study baseline mean age**

**
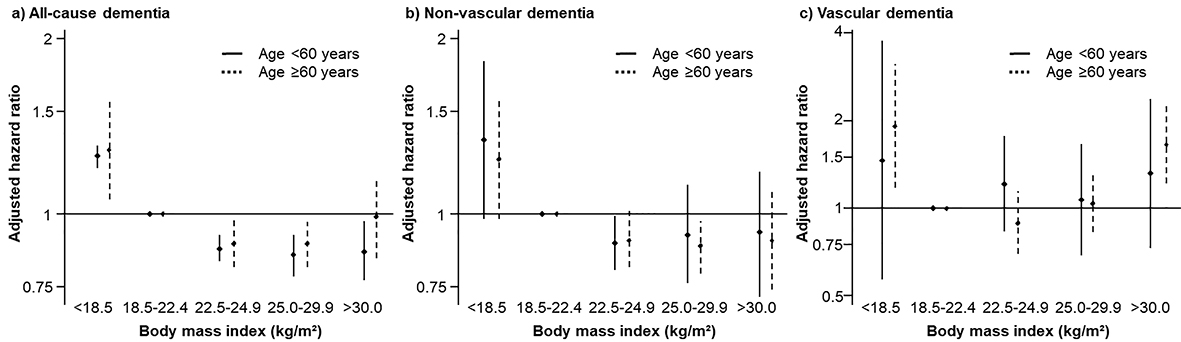
**

Hazard ratios adjusted for age, smoking, and education or socioeconomic status

**Fig. S9: Associations between dementia and body mass index by study design**

**
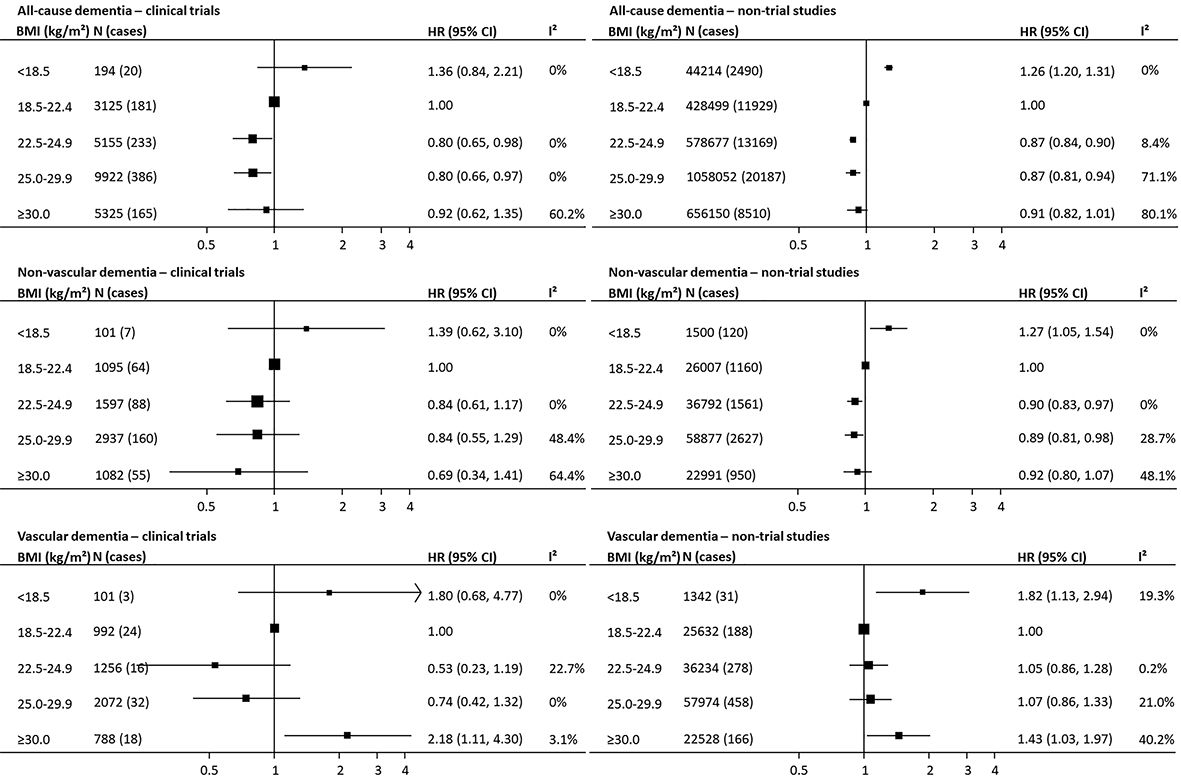
**

Hazard ratios adjusted for age, smoking, and education or socioeconomic status

**Fig. S10: Random effects pooled age adjusted hazard ratios with 95% confidence intervals of all-cause dementia for second fifth of waist circumference with the first fifth of waist circumference as referent**

**Fig. S11: Random effects pooled age adjusted hazard ratios with 95% confidence intervals of all-cause dementia for third fifth of waist circumference with the first fifth of waist circumference as referent**

**Fig. S12: Random effects pooled age adjusted hazard ratios with 95% confidence intervals of all-cause dementia for fourth fifth of waist circumference with the first fifth of waist circumference as referent**

**Fig. S13: Random effects pooled age adjusted hazard ratios with 95% confidence intervals of all-cause dementia for last fifth of waist circumference with the first fifth of waist circumference as referent**


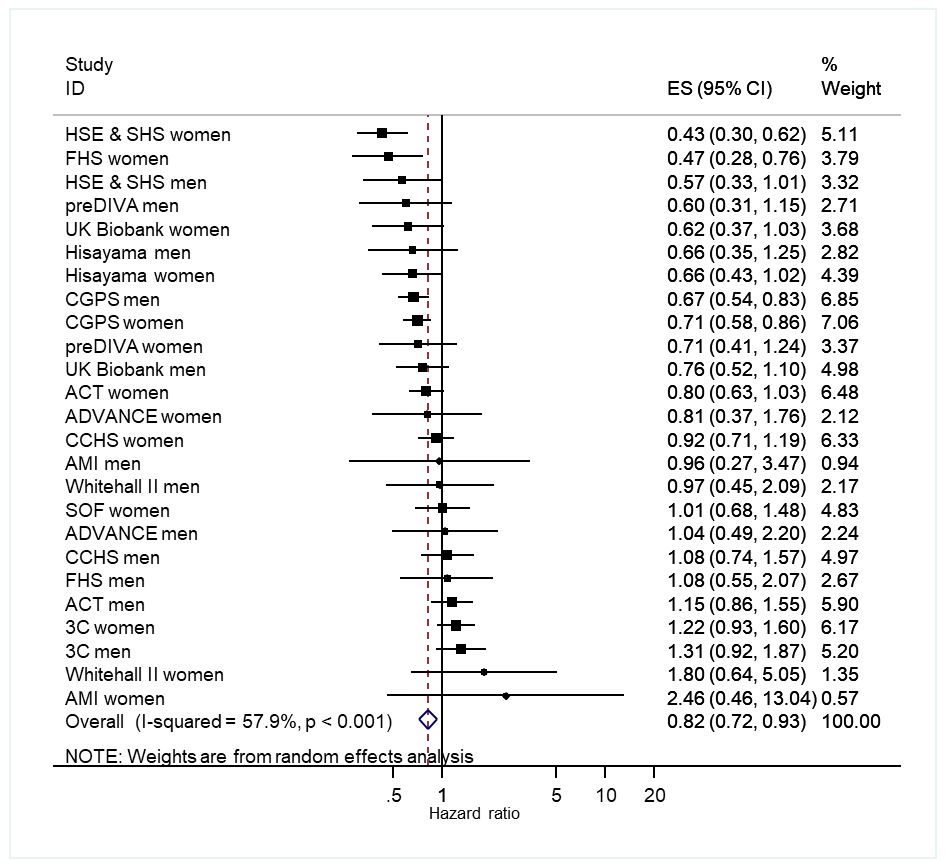


**Fig. S14: Associations between all-cause dementia and waist circumference by dementia ascertainment**


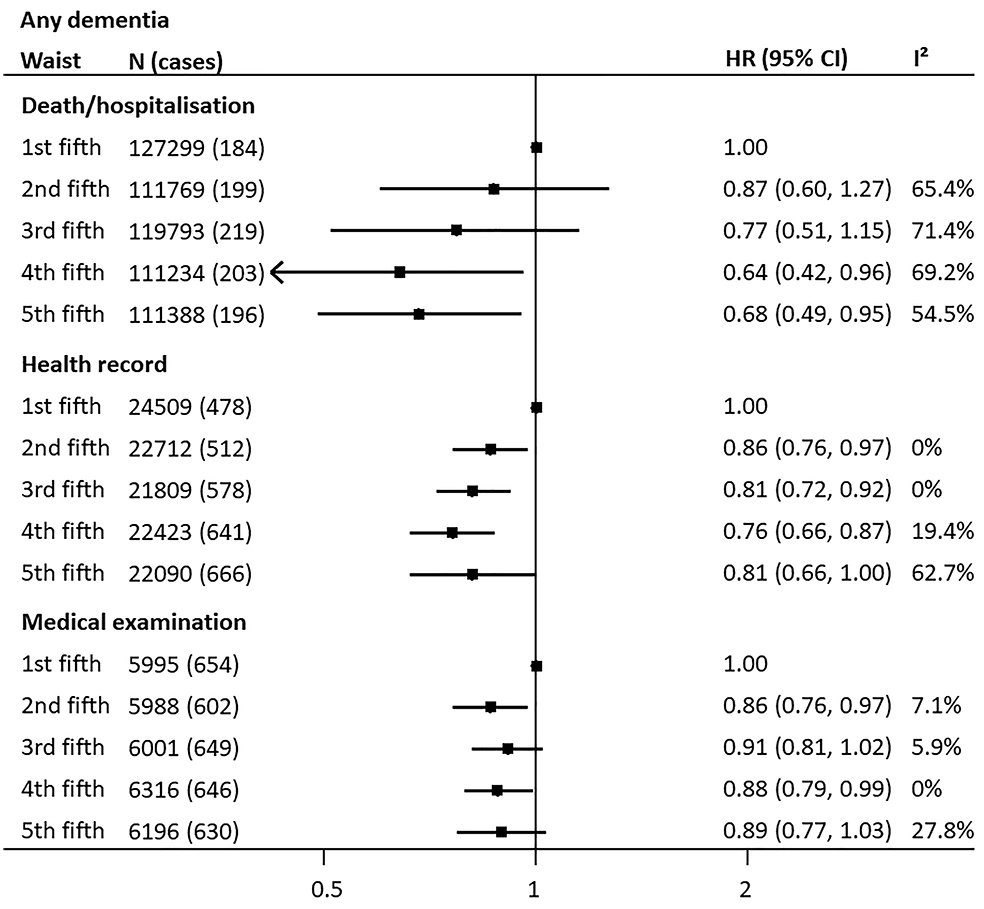


Hazard ratios adjusted for age, smoking, and education or socioeconomic status

**Fig. S15: Associations between dementia and waist circumference by study design**


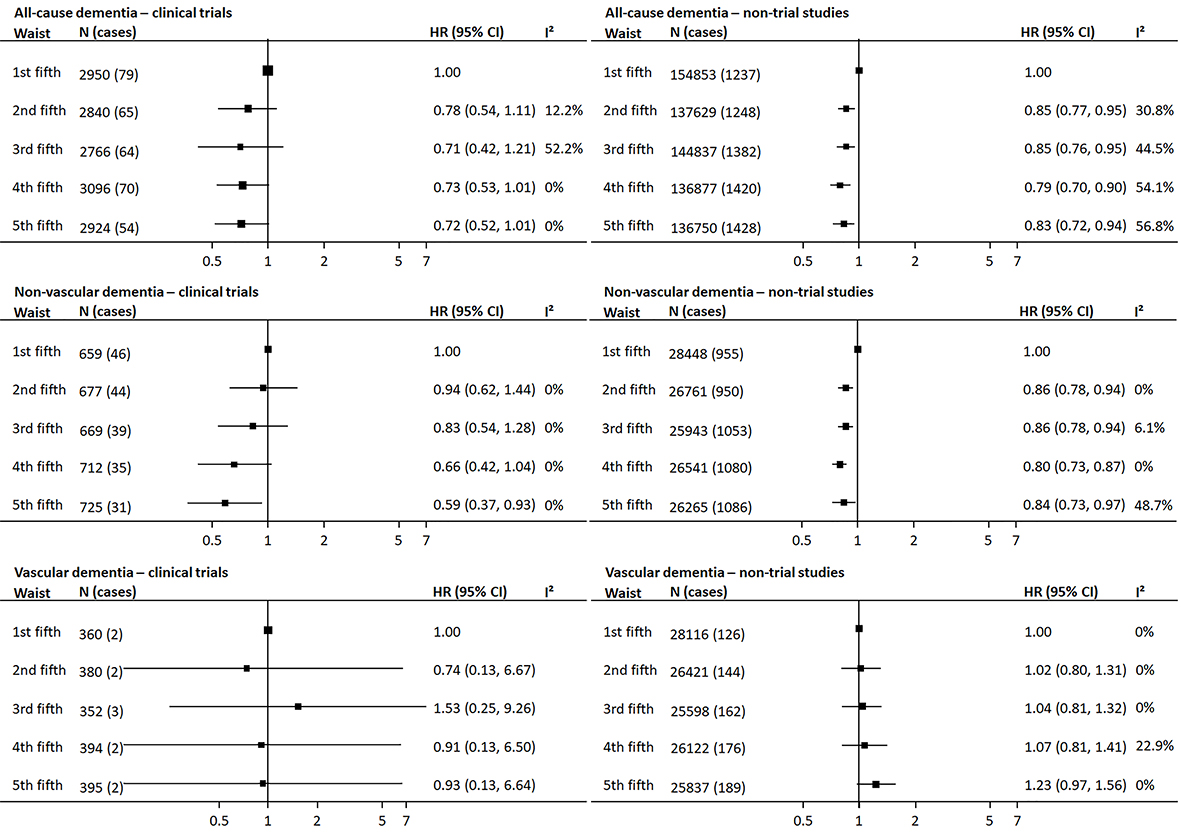


Hazard ratios adjusted for age, smoking, and education or socioeconomic status

**Fig. S16: Associations between dementia and waist circumference by study baseline mean age**

**
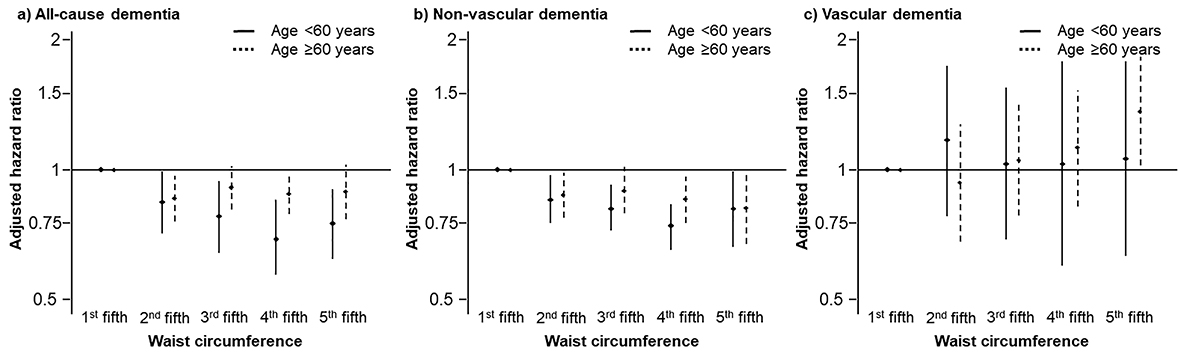
**

Hazard ratios adjusted for age, smoking, and education or socioeconomic status

**Fig. S17: Random effects pooled age adjusted hazard ratios with 95% confidence intervals of all-cause dementia for ≥0.5% weight loss per year with <0.5% weight change has referent**

**Fig. S18: Random effects pooled age adjusted hazard ratios with 95% confidence intervals of all-cause dementia for ≥0.5% weight gain per year with <0.5% weight change has referent**

**Fig. S19: Associations between all-cause dementia and annual percent weight change by study baseline mean age**

**
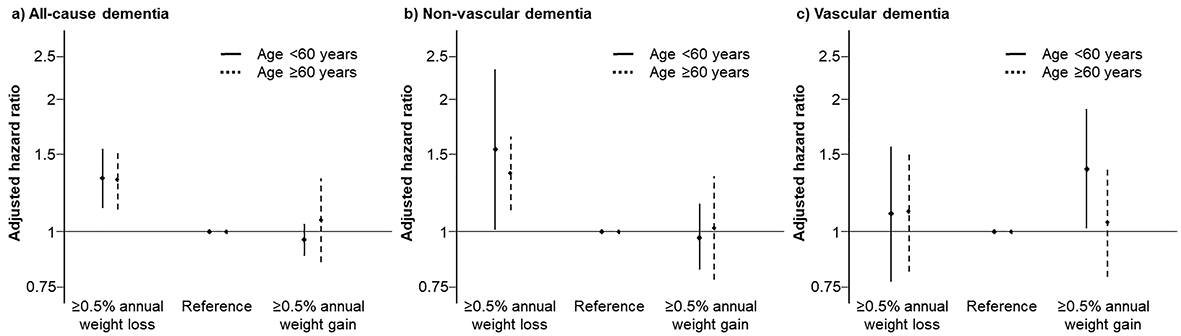
**

Hazard ratios adjusted for age, smoking, and education or socioeconomic status

**Fig. S20: Associations between all-cause dementia and annual percent weight change by dementia ascertainment**


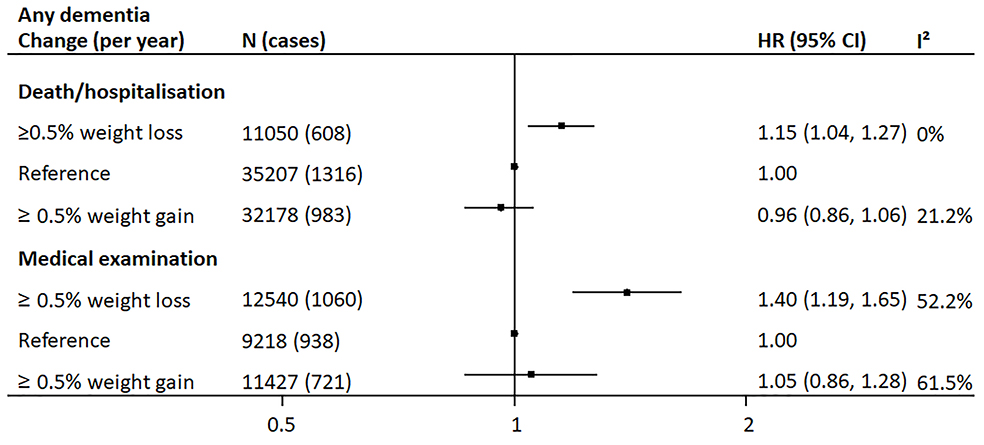


Hazard ratios adjusted for age, smoking, and education or socioeconomic status
